# DYNAMEX: A Time-Resolved Model for Predicting Distribution of Chemicals in In Vitro Cell Assays

**DOI:** 10.1021/acs.est.6c06087

**Published:** 2026-08-26

**Authors:** Fabian C. Fischer, Liam Geyer, Miguel Rodriguez, Luise Henneberger, Beate I. Escher

**Affiliations:** † Department of Pathology and Laboratory Medicine, Brown University, Providence, Rhode Island 02912, United States; ‡ Department of Biomedical and Pharmaceutical Sciences, 4260 University of Rhode Island, Kingston, Rhode Island 02881, United States; § Helmholtz Centre for Environmental Research - UFZ, 28342 Department Cell Toxicology, Permoserstraße 15, Leipzig 04318, Germany; ∥ Eberhard Karls University Tübingen, Department of Geosciences, Tübingen 72074, Germany

**Keywords:** dosimetry, quantitative in vitro−in vivo extrapolation, serum-mediated passive dosing, PFAS, cellular uptake kinetics

## Abstract

High-throughput in vitro cell assays are promising tools for predicting chemical-induced health effects in humans. Quantifying freely dissolved medium (*C*_free,medium_) and cellular concentrations is essential for robust quantitative in vitro–in vivo extrapolation (QIVIVE), but the miniaturized format of 384- and 1536-well plates makes these metrics challenging to measure directly. We developed DYNAMEX (dynamic NAM exposure), a time-resolved kinetic model simulating chemical fate in cell assays, accounting for volatilization, medium binding, well-plate sorption, and cellular uptake, including growth dilution. The model accurately captured uptake kinetics for neutral compounds and newly measured PFAS cellular uptake kinetics, reproduced empirical thresholds for volatilization losses, and predicted free fractions in medium across 51 compounds (RMSE = 0.49 log_10_ units) and cellular-to-nominal concentration ratios across 17 compounds (RMSE = 0.56 log_10_ units) under different bioassay conditions. Simulations across 113 chemicals and varying assay setups showed that, under standard assay conditions using 10% FBS, equilibrium mass balance modeling predicted *C*_free,medium_ within 10% for 83 of 113 compounds and is sufficient for most applications. Kinetic modeling is required when volatilization or well-plate sorption causes substantial mass losses, particularly under serum-free conditions and in miniaturized formats, or when low membrane permeability limits cellular uptake within the assay duration, as observed for several hydrophobic ionizable organic chemicals. The model provides a mechanistic framework to improve in vitro dosimetry, guide assay design, and support integration of time-resolved exposure metrics into QIVIVE and in vivo modeling workflows.

## Graphical abstract

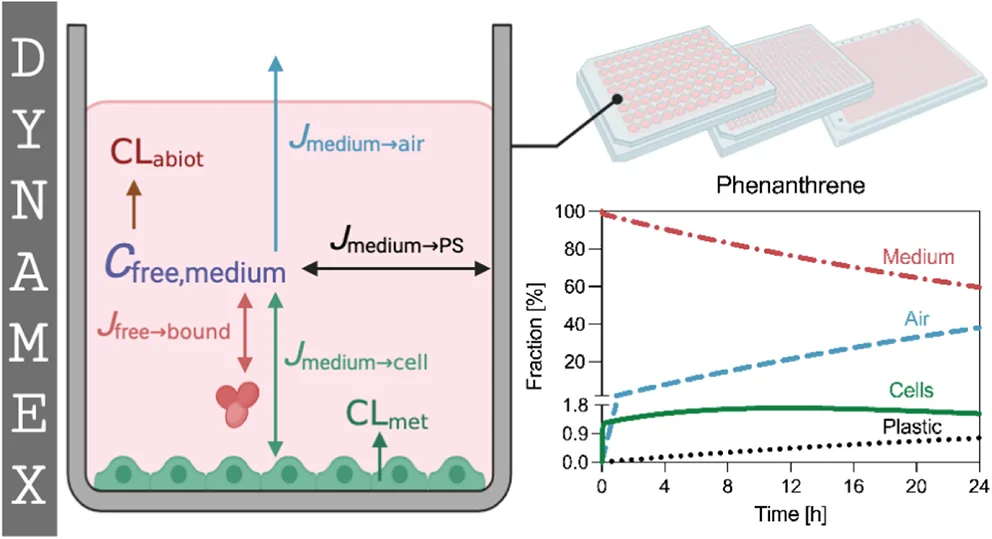

## 1 Introduction

The growing number and structural diversity of manufactured chemicals demand efficient testing strategies to evaluate potential human health effects. High-throughput in vitro assays using cultured mammalian cell lines offer a promising alternative to animal testing for chemical risk assessment.^1^ Reporter gene assays using recombinant cells with fluorescent or luminescent readouts have been developed to visualize chemical interactions with molecular targets.^2^ Successful application of high-throughput in vitro assays in chemical risk assessment requires improved translation of in vitro effect concentrations to human exposure conditions.^3^ Quantitative in vitro to in vivo extrapolation (QIVIVE) is most effectively performed by comparing free concentrations in the assay medium (*C*_free,medium_) or cells (*C*_free,cell_) with corresponding *C*_free_ in human blood or target tissues.^4^ While low medium volumes impede routine measurements of *C*_free,medium_, solid-phase microextraction (SPME)-based methods are available for 96- and 384-well plates,^5–9^ and SPME methods can also be employed for blood.^10,11^

In vitro cell assays have been increasingly miniaturized over recent decades to enhance high-throughput screening (HTS) capabilities using robotic platforms. Standard formats now employ 96- to 1536-well plastic plates with 6–200 μL of exposure medium, small cell populations (1,000–20,000 cells), and short assay durations (2–24 h), enabling automated testing of thousands of chemicals. For example, the HTS screening program *Tox21* fingerprinted 10,000 chemicals in ∼60 reporter gene assays at nominal concentrations (*C*_nom_) ranging from 1 nM to 100 μM.^12–14^ In Tox21, *C*_free,medium_ and *C*_free,cell_ were not quantified, volatile chemicals were not excluded, and the dosed concentration range was fixed. In vitro distribution models are therefore needed to retrospectively assess the validity of the reported effect concentrations. For nonvolatile chemicals, these models also allow QIVIVE input parameters to be estimated from nominal effect concentrations. These models can also be used to plan experimental dosing ranges that avoid oversaturation of the medium^15^ and baseline membrane toxicity,^16^ and to exclude volatile chemicals from experiments in conventional setups.^17^

Numerous equilibrium partitioning and empirical models have been developed to estimate *C*_free,medium_ from *C*_nom._ ^18–22^ These models typically predict chemical distribution based on partition coefficients (*K*) and the volumes of system compartments. Equilibrium models assume instantaneous distribution between compartments such as medium, air, cells, and well plastic. They further assume that *C*_free,medium_ equals *C*_free,cell_. *C*_free,cell_ is the biologically effective dose for targets in the cytosol once steady state is reached and in direct contact with molecular targets in membranes and organelles.^23^ However, slow cell uptake kinetics might prevent that equilibrium is reached between *C*_free,medium_ and *C*_free,cell_ within typical assay durations, especially for ionizable organic chemicals (IOCs) with low membrane permeabilities.^24–27^ Time-resolved in vitro exposure models are therefore needed to assess temporal dynamics of *C*_free,medium_ and *C*_free,cell_ to optimize assay design for QIVIVE.

Our previously published mass balance model demonstrated that fetal bovine serum (FBS), a nutrient-rich supplement commonly used in cell culture media, constitutes the dominant sorptive phase in plate-based cell assays.^20^ Although FBS can reduce apparent assay sensitivity,^28–31^ desorption of FBS-bound chemicals buffers against depletive losses to volatilization,^17^ well plate sorption,^32^ cellular uptake,^27^ cellular metabolism,^33^ and abiotic degradation,^34^ thereby maintaining stable *C*_free,medium_ over time. This concept of serum-mediated passive dosing has been experimentally validated for assays containing sufficiently high FBS concentrations.^5,27,35,36^ In contrast, serum-free or low-FBS conditions^37,38^ reduce the sorptive capacity of the medium, amplifying the impact of depletion processes.

The kinetic processes that need to be considered in in vitro assays, including volatilization loss,^36^ sorption to well-plate materials,^31,32^ cellular uptake kinetics,^21^ abiotic degradation,^34^ cellular metabolism,^33^ and dilution due to cell proliferation, have each been experimentally characterized or modeled in isolation.^18,20,21^ Two recent models integrate these processes into a single time-resolved framework. The fugacity-based IV-MBM DP model of Bloch et al. resolves chemical distribution over time from physicochemical properties, adding repeated dosing and facilitated transport across the UWL, but represents cellular uptake through partitioning and boundary-layer transport rather than explicit membrane permeability.^39^ The INSIGHT model uses a kinetic framework calibrated across 42 chemicals and 7 cell lines using Bayesian inference,^40^ but requires cell line-specific calibration of partition coefficient correction factors and net efflux rates, which limits its extension to cell lines and chemicals outside the calibration set. Here, we present DYNAMEX (dynamic NAM exposure), a fully mechanistic, time-resolved exposure model that integrates the same processes from first-principles, without compound- or cell line-specific fitting. The model explicitly resolves intrinsic membrane permeability, unstirred water layer (UWL) transport, and cell proliferation as kinetic processes that can sustain disequilibrium between *C*_free,medium_ and *C*_free,cell_, and includes an optional parameter fitting tool to estimate intrinsic kinetic parameters from experimental data.

Differential equations were solved numerically in *R*, with the full code provided for user application and modification. Model performance was evaluated by comparing simulated temporal *C*_free,medium_ and total cellular concentrations (*C*_cell_) against experimental data from in vitro assays. The data include newly measured cellular uptake kinetics of four PFAS, which to our knowledge represent the first time-resolved uptake data for these compounds in in vitro cell assays. Simulations were extended to 113 neutral and ionizable chemicals covering 7 orders of magnitude in lipophilicity expressed as octanol–water partition coefficient *K*_ow_ (−0.07 < log *K*_ow_ < 7.7) to evaluate dynamics of exposure across different FBS contents, cell lines, and well plate formats. Finally, we discuss how this model can support in vitro dosimetry and enable the use of predicted *C*_free,medium_ and *C*_free,cell_ in QIVIVE frameworks.

## 2 Model Theory

### 2.1 Model Overview

The DYNAMEX model accounts for (1) loss to the headspace, (2) binding to and desorption from proteins and lipids in the medium, (3) abiotic degradation in the medium, (4) diffusion into well plate plastics, (5) cellular uptake, (6) cell proliferation and growth dilution, (7) intracellular distribution, and (8) cellular metabolism (Figure 1). The model assumes that only freely dissolved (i.e., unbound) chemicals move between compartments.^41^ As *C*_free,medium_ declines due to partitioning and loss processes, the model simulates compensatory desorption from proteins and lipids in the medium. Temporal changes in *C*_free_ and bound concentrations in both cells (*C*_bound,cell_) and medium (*C*_bound,medium_) are calculated, along with the area under the curve (AUC) of concentration–time profiles, time to reach 95% of intracellular equilibrium (*t*_95%_), and percentage losses attributable to well plate sorption, abiotic degradation, cellular metabolism, and volatilization.

**Figure 1:**
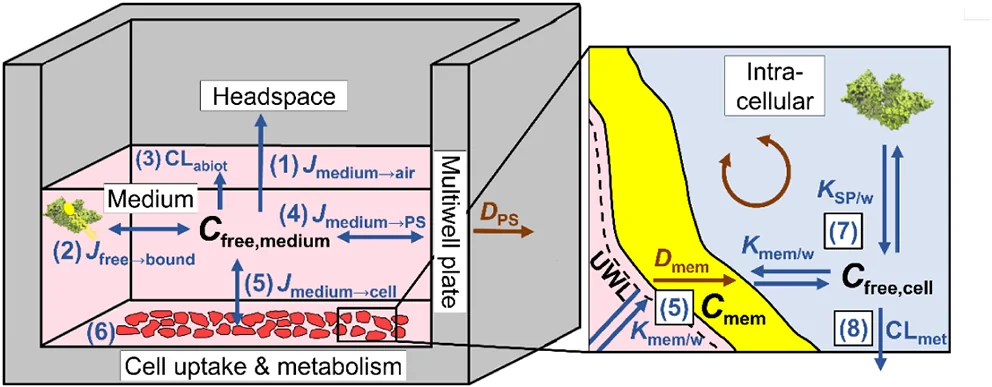
Illustrative scheme of the *in vitro* test system with partitioning, diffusion, and elimination processes included in the model and chemical input parameters required for the simulation. The numbers refer to the numbered processes in Section 2.1.

The medium and cell interior are treated as well-mixed aqueous phases, assuming instantaneous mixing of freely dissolved chemicals and no explicit spatial resolution within the cell. Flux of freely dissolved chemical from the medium into the cells is modeled as a single permeability-limited uptake process governed by the apparent permeability (*P*_app_, cm/s) that combines resistances from diffusion across the UWL adjacent to the cell surface and permeation across the cell membrane (eqs S3–S5), without resolving transmembrane and intracellular gradients. This simplification avoids the need for a three-dimensional model assuming that freely dissolved intracellular concentrations equilibrate rapidly following membrane transport. The model accounts for facilitated transport by medium protein-bound chemicals across the UWL.^42–44^

To describe uptake into the well plate, we modeled chemical transport as one-dimensional (1D) diffusion into stacked layers of well-plate plastic. This approximation assumes that net 1D flux reasonably captures overall uptake into the three-dimensional well geometry. Although the well plate is modeled as a large sorptive reservoir, diffusion between the medium and plastic is bidirectional, allowing back-diffusion into the medium when *C*_free,medium_ declines due to competing loss processes. In contrast, volatilization to the headspace is modeled unidirectionally, assuming a well-ventilated air volume that does not permit back-diffusion although it is known that volatile chemicals can contaminate adjacent wells.^17^

Pre-equilibration of the test compound in the medium prior to its addition to the well plate, as it is common practice in cell-based bioassays, was assumed to establish equilibrium between medium colloids and the aqueous phase at the start of the assay.^15^ Reversible binding to medium proteins and lipids is included to reflect the stabilizing effect of serum-mediated passive dosing on *C*_free_.^15,20^ Chemical distribution between compartments occurs via diffusion and interfacial partitioning, governed by concentration gradients. Bidirectional chemical fluxes (*J*) are defined by these gradients. For example, the net flux from medium to cells (*J*_free,medium→free,cell_) is positive when *C*_free,medium_ exceeds *C*_free,cell_, and reverses once the gradient is inverted. The equations governing temporal changes in *C*_free,medium_, *C*_bound,medium_, *C*_free,cell_, *C*_bound,cell_, and well concentrations (*C*_well_) based on fluxes within and between compartments are presented in the following sections.

### 2.2 Temporal Concentration Changes

At the start of the simulation, the chemical is assumed to be fully dissolved and uniformly mixed within the medium, while concentrations across all spatial layers *x* in all nonmedium compartments are set to zero:

1 C(x,t=0)=0for allx

Temporal changes in concentration (*dC/dt*) are then calculated by summing the chemical fluxes *J* (mol/s) within and between system compartments.

#### 2.2.1 Medium Concentrations

Temporal changes in *C*_free,medium_ (*dC*_free,medium_/*dt*, mol/mL_w_/s) are governed by desorption from medium proteins and lipids (*J*_bound,medium→free,medium_), uptake into cells (*J*_free,medium→free,cell_), diffusion into the well plate (*J*_free,medium→well_), volatilization to the headspace (*J*_free,medium→air_), and abiotic degradation in the medium (*J*_free,medium→AD_):

2 $$\frac{dC_{\mathrm{free},\mathrm{medium}}}{dt}=\frac{J_{\mathrm{bound},\mathrm{medium}\to \mathrm{free},\mathrm{medium}}-J_{\mathrm{free},\mathrm{medium}\to \mathrm{free},\mathrm{cell}}-J_{\mathrm{free},\mathrm{medium}\to \mathrm{well}}-J_{\mathrm{free},\mathrm{medium}\to \mathrm{air}}-J_{\mathrm{free},\mathrm{medium}\to \mathrm{AD}}}{V_{w,\mathrm{medium}}}$$

*V*_w,medium_ is the volume of water in the medium (mL_w,medium_).

Temporal changes in bound concentrations in the medium (*dC*_bound,medium_/*dt*, mol/mL_prot+lip,medium_/s) depend on *J*_bound,medium→free,medium_:

3 $$\frac{dC_{\mathrm{bound},\mathrm{medium}}}{dt}=\frac{-J_{\mathrm{bound},\mathrm{medium}\to \mathrm{free},\mathrm{medium}}}{V_{\mathrm{prot}+\mathrm{lip},\mathrm{medium}}}$$

*V*_prot+lip,medium_ is the sum of sorptive colloids (proteins and lipids) in the medium (mL_prot+lip,medium_).

#### 2.2.2 Cell Concentrations

Temporal changes in *C*_free,cell_ (*dC*_free,cell_/*dt*, mol/mL_w,cell_/s) depend on the desorption of chemicals from cell proteins and lipids (*J*_bound,cell→free,cell_), uptake into cells (*J*_free,medium→free,cell_), cell metabolism (*J*_free,cell→met_), and cell growth (*dV*_w,cell_/*dt*):

4 $$\frac{dC_{\mathrm{free},\mathrm{cell}}}{dt}=\frac{J_{\mathrm{bound},\mathrm{cell}\to \mathrm{free},\mathrm{cell}}+J_{\mathrm{free},\mathrm{medium}\to \mathrm{free},\mathrm{cell}}-J_{\mathrm{free},\mathrm{cell}\to \mathrm{met}}}{V_{w,\mathrm{cell}}}-C_{\mathrm{free},\mathrm{cell}}\cdot \frac{\frac{dV_{w,\mathrm{cell}}}{dt}}{V_{w,\mathrm{cell}}}$$

*V*_*w,cell*_ is the time-dependent volume of water in the cells (mL_w,cell_), calculated dynamically from eq 6 to account for cell proliferation over the course of the assay.

Temporal changes in *C*_bound,cell_ (*dC*_bound,cell_/*dt*, mol/mL_prot+lip,cell_/s) depend on *J*_bound,cell→free,cell_ and cell growth (*dV*_prot+lip,cell_/*dt*):

5 $$\frac{dC_{\mathrm{bound},\mathrm{cell}}}{dt}=\frac{-J_{\mathrm{bound},\mathrm{cell}\to \mathrm{free},\mathrm{cell}}}{V_{\mathrm{prot}+\mathrm{lip},\mathrm{cell}}}-C_{\mathrm{bound},\mathrm{cell}}\cdot \frac{\frac{dV_{\mathrm{prot}+\mathrm{lip},\mathrm{cell}}}{dt}}{V_{\mathrm{prot}+\mathrm{lip},\mathrm{cell}}}$$

*V*_prot+lip,cell_ is the sum of sorptive proteins and lipids in the cells (mL_prot+lip,cell_).

Equations 4 and 5 incorporate cell growth-induced dilution by adjusting *C*_free,cell_ and *C*_bound,cell_ according to the time-dependent expansion of intracellular water (*V*_w,cell_, mL_w,cell_) and *V*_prot+lip,cell_. As cells grow, increases in volume lead to proportional decreases in concentration, preserving mass balance. Cell growth is modeled as a linear process, with *V*_w,cell_ and *V*_prot+lip,cell_ increasing at constant rates from their initial values to experimentally determined maximum values (*V*_w,cell,max_ and *V*_prot+lip,cell,max_):

6 $$\frac{dV_{w,\mathrm{cell}}}{dt}=\left(\mathrm{GF}-1\right)\cdot \frac{V_{w,\mathrm{cell},0}}{t_{\mathrm{assay}}}$$

7 $$\frac{dV_{\mathrm{prot}+\mathrm{lip},\mathrm{cell}}}{dt}=\left(\mathrm{GF}-1\right)\cdot \frac{V_{\mathrm{prot}+\mathrm{lip},\mathrm{cell},0}}{t_{\mathrm{assay}}}$$

The maximum cell volumes were set to *V*_cell,max_ = GF × *V*_cell,0_, where *V*_cell,0_ is the initial cell volume (mL) and the growth factor (GF) is the fold increase in cell number over the assay duration (*t*_assay_).

#### 2.2.3 Distribution Ratios of Ionizable Chemicals

Distribution equilibrium is modeled as the weighted sum of neutral and ionized species at pH 7.4, with distinct partition coefficients between phase 1 and phase 2 (*K*_1/2_) assigned to each chemical species based on their fraction at assay pH 7.4.^45^ The overall distribution ratio (*K*) is calculated as

8 $$K_{1/2}=f_{\mathrm{neutral}}\cdot K_{1/2,\mathrm{neutral}}+\sum f_{\mathrm{ion}}\cdot K_{1/2,\mathrm{ion}}$$

∑*f*_ion_·*K*_ion_ = 0 is assumed for diffusion into the well plate^46^ and volatilization.^47^ The assay pH of 7.4 is a user-defined input. For example, medium-water and cell-water partition coefficients (*K*_prot+lip,medium/w_, *K*_prot+lip,cell/w_), or the individual proteins and lipids constructing *K*_prot+lip,medium/w_ and *K*_prot+lip,cell/w_ in the model (eqs S9 and S12), and *P*_app_ can be supplied at different pH to represent a shifted medium pH or a lower intracellular pH, with *P*_app_ measured in Transwell experiments under an apical-to-basolateral pH gradient.

#### 2.2.4 Chemical Fluxes between and within Compartments

The equations describing the chemical fluxes between compartments (medium to cells, well plate, and headspace) and within compartments (diffusion in plastic plates, protein and lipid binding in medium and cells, abiotic degradation, and cellular metabolism) are presented in Sections S1 and S2 of the Supporting Information (eqs S1–S17).

### 2.3 Computation in *R*

The *R* code for modeling distribution of organic chemicals in an in vitro cell assay loads system and chemical parameters via an Excel workbook and is structured into five sections:

Section R1 defines the system parameters, including volumes, interface areas, and volume fractions of proteins, lipids, and water in the medium and cells.

Section R2 outlines the chemical parameters: Molecular weight, partition coefficients, diffusion coefficients, desorption rate constants, metabolic and degradation rate constants.

Section R3 defines initial conditions.

Section R4 contains the function that solves the differential equations described in Sections 2.1, S1, and S2 numerically. The *R* function uses the *ODE.solver*, specifically *lsoda*, which can dynamically switch between integration methods for stiff and nonstiff systems with vastly different or uniformly scaled transport rates.^48^ The function calculates temporal concentrations in the medium and cells over time in dynamic time steps (*dt*), and for diffusion in well plate plastic, at dynamic time and discrete spatial steps (*dt*, *dx*). The *lsoda* solver decreases *dt* when the model calculates rapid fluxes in or between compartments (e.g., high diffusion rates or steep concentration gradients) to maintain accuracy while increasing *dt* when mass transfer is slower to optimize the balance between computational efficiency and model precision.

Section R5 computes the processing, extraction, plotting, and analysis of the simulated data. Furthermore, mass balance calculations are presented to verify flux consistency and to assess any potential losses due to numerical errors.

Additionally, we provide a standalone parameter fitting tool (*Parameter fit model.R*) as a supplementary *R* script that wraps the ODE solver in a bounded nonlinear optimizer (*nlminb*) to estimate intrinsic model parameters that are challenging to measure directly in vitro. Chemical and system parameters, observed time-course data, and fitting settings are user-defined via an Excel workbook. The fittable parameters are intrinsic metabolic clearance (CL_met_), abiotic clearance (CL_abiot_), and membrane permeability (*P*_mem_). *P*_mem_ differs from *P*_app_ commonly reported in permeability assays, which can be confounded by the UWL and protein-facilitated transport. The fit model derives *P*_app_ internally from the fitted *P*_mem_ and the calculated UWL resistance. An optional extension supports time-dependent metabolic clearance, where CL_met_(*t*) = CL_met,0_ × (1 + *r*_ind_ × *t*), with the induction ratio *r*_ind_ = *m*_ind_/*M*_control_ derived by linear regression from user-supplied metabolic activity data, where *M*_control_ is the basal metabolic activity and *m*_ind_ is the induction rate. Fit optimization is performed on log-transformed parameters, with residuals normalized by the mean observed value to give equal weight across variables of different magnitudes. Observed data can include any combination of total or free concentrations in the medium or cells. Reliable identification of an intrinsic rate requires time points that resolve both the uptake phase and the approach to steady state. We recommend at least five to six time points across these phases, with at least two replicates per point. Fitted parameter values are reported with 95% confidence intervals derived from the numerical Hessian, alongside goodness-of-fit metrics (RMSE, RMSLE, and average fold error) for each fitted variable.

## 3 Model Parameterization and Experiments

We parameterized the model for standard 2D high-throughput assays and for 132 chemicals with diverse physicochemical properties. All system and chemical parameters and sources are provided in Tables S1–S8 of the Supporting Information with details on their derivation below.

### 3.1 Chemical Parameters

Partition coefficients to phospholipid vesicles (liposomes, lip), proteins (bovine serum albumin, BSA) and structural proteins (SP) were compiled and are listed in Tables S1 and S2.^49–54^ Partition coefficients for chemicals without experimental data were predicted using polyparameter linear free energy relationships (pp-LFERs), as described elsewhere.^52,53,55^ The diffusion of chemicals in the plastic of the multiwell plate was estimated using the mean diffusion coefficient in polystyrene (*D*_PS_) of 2.33 × 10^–16^ cm^2^ s^–1^, as measured for 20 neutral chemicals.^32^ Air–water partitioning (*K*_aw_) of neutral chemicals and neutral species of IOCs was preferably calculated using experimentally determined Henry constants at 25 °C from the literature. For chemicals with no experimental data, *K*_aw_ was estimated using pp-LFERs at 37 °C. The reference temperature of each parameter is listed in Tables S1 and S2. Parameters available only below the assay temperature of 37 °C, at 22 or 25 °C, were used without temperature correction. The enthalpies of phase transfer required for such a correction were not available, and for partitioning between two condensed phases the correction is small and offset by the uncertainty of the predictions.^47^ The Henry constant is the exception, typically increasing by up to a factor of 4 (0.6 log units) over this range.^47^

We estimated the desorption rates of neutral and ionizable chemicals from medium and cell proteins and lipids to water based on *k*_des_ measured for desorption of neutral chemicals from BSA at 37 °C, which demonstrated a strong inverse correlation with the molecular weight of the chemicals:^56^

9 $$k_{\mathrm{des}}\left(37^{{}^{\circ}}C\right)=20267\cdot {\mathrm{MW}}^{-2.0}$$

*K*_well/w_ were derived for polystyrene multiwell plates. Polystyrene-water partition coefficients (*K*_PS/w_, mL_w_/mL_PS_) of neutral chemicals were previously shown to correlate with the log *K*_ow_ of chemicals,^32^ and this relationship was used for prediction of *K*_PS/w_:

10 $$\log K_{\mathrm{PS}/w}=0.56\cdot \log K_{\mathrm{ow}}-0.05$$

The diffusion coefficient in the aqueous phase of the medium (*D*_w_, cm^2^ /s) at 37 °C was predicted from the molecular weight of the test chemical (g/mol):^57^

11 $$D_{w}=9.9\times 10^{-5}\cdot {\mathrm{MW}}^{-0.453}$$

The diffusion coefficient in the air (*D*_air_, cm^2^ s^–1^) at 37 °C was estimated from *D*_w_:

12 $$D_{\mathrm{air}}=10^{5}\cdot D_{w}$$

The diffusion coefficient across the membrane *D*_mem_ at 37 °C was predicted from the molecular weight of the chemical:^24^

13 $$D_{\mathrm{mem}}=10^{-5.13-0.453\cdot \log\left(\mathrm{MW}\right)}$$

The diffusion coefficient for albumin in water (*D*_alb_, cm^2^ s^–1^) as a parameter for medium protein-facilitated transport was derived from the literature.^58^ As no comparable prediction method is available for IOCs, we compiled experimental *P*_app_ of IOCs reported in various permeability assays (Table S1). Values measured under shaking conditions were preferred, because minimizing the UWL contribution means the measured *P*_app_ approaches the intrinsic *P*_mem_. The model then calculates an independent UWL resistance based on the conditions of the modeled cell assay (eq S3), which may lead to a conservative estimate of overall transport resistance for compounds where the experimental *P*_app_ retain residual UWL contribution.

### 3.2 System Parameters

#### 3.2.1 Well Plate Geometry and Medium Volumes

To derive the well plate geometry and medium volumes used in typical in vitro assays, we accessed the Corning product selection guide.^59^ The guide provides information on typical media volumes, the surface areas of well plastic in contact with the medium, and the thickness of the plastic at the bottom and walls of the well plate. Using these values, we calculated the medium volume to plastic surface area ratios. All well plate dimensions and corresponding medium volumes are reported in Table S3.

#### 3.2.2 Protein and Lipid Composition of Cells and Media

Data on the protein, lipid, and water content of cells and media components (base medium, serum) were collected from the literature.^7,20^ In addition, we measured protein and lipid content of 15 widely used medium constituents using the Lowry and the sulfo-phosphovanillin assays, respectively, as previously described^20^ and detailed in Section S3. All protein, lipid, and water contents of cells and media are reported in Tables S4 and S5. The cell GFs are reported in Tables S6 and S7, from which maximum cellular volumes were derived (eqs 6 and 7). These cell growth parameters and assumption of linear cell growth were derived from specific cell seeding densities and should be applied with caution if the seeding density or cell line differs. Specifically, if confluence is increasing above 80% contact inhibition will slow down the pseudolinear growth and reach saturation.^60^

BSA was used as a surrogate for proteins in the assay medium, while structural proteins such as those found in muscle tissue were used to represent intracellular proteins. Phospholipid liposomes were employed as proxies for lipid constituents in both medium and cells. Therefore, the model requires bovine serum albumin-water (*K*_BSA/w_), structural protein–water (*K*_SP/w_), and liposome-water (*K*_lip/w_) partition coefficients.

#### 3.2.3 Cell Morphology

The surface area for each cell type was calculated based on their dimensions as reported in various sources (Table S8). We assumed a spherical shape for simplicity, using the formula for the surface area of a sphere, 4πr^2^, where r is the cell radius.

### 3.3 Cellular Uptake Kinetics of PFAS

Uptake kinetics of four PFAS (perfluorooctanoic acid (PFOA), perfluorooctanesulfonic acid (PFOS), perfluorononanoic acid (PFNA), and perfluorododecanoic acid (PFDoA)) were measured in HepG2 cells (3.0 × 10^5^ cells/well, 1 mL DMEM with 0.5% FBS) over 48 h. Media and cells were harvested at nine time points and analyzed by UHPLC-QTOF-MS/MS following EPA Method 1633.^61^ Cell growth was quantified from phase-contrast microscopy images using a custom *R* workflow. Full experimental details are provided in Section S4.

## 4 Results and Discussion

### 4.1 Model Evaluation for Neutral Chemicals

Cellular uptake kinetics were evaluated for previously published data of the neutral compounds benzo­[a]­pyrene (BaP) and benzo­[ghi]­perylene (BghiP). Simulations were run in 96-well plates containing 120 μL of DMEM with 0.5% FBS and 16,000 HEK293 cells to replicate assay conditions used in Fischer et al.^27^ Metabolic enzyme measurements showed that HEK293 cells were not metabolically active,^33^ so CL_met_ was set to 0. Modeled intracellular uptake kinetics closely reproduced the experimental time course for both compounds (Figure S1). For BghiP, the modeled and measured *t*_95%_ were comparable (20 h modeled vs 13 h measured). For BaP, the model overestimated the approach to equilibrium (*t*_95%_: 5.0 h modeled vs 2.8 h measured), but accurately captured the observed uptake trajectory over the experimentally resolved time window (Figure S1A).

Model performance under varying medium protein content was evaluated by simulating experimental conditions from Fischer et al.,^27^ using 96-well plates seeded with 9,000–16,000 HEK293 cells in DMEM supplemented with 0.5–10% FBS and experimental cell growth rates (Table S6). Both measured cellular fluorescence intensities (FI_cell_) and modeled *C*_cell_ of BaP declined with increasing FBS (Figure 2A). From 0.5% to 10% FBS, experimental FI_cell_ decreased by 72%, while simulated *C*_cell_ decreased by 78%. Simulated *t*_95%_ values declined from 5.0 h at 0.5% FBS to 2.0 h at 10% FBS, while experimental *t*_95%_ values decreased from 3.3 ± 0.4 h to 1.4 ± 0.3 h over the same range. Fold-differences between simulated and measured *t*_95%_ were 1.5 (0.5% FBS), 1.4 (1% FBS), 1.7 (2% FBS), 1.6 (5% FBS), and 1.4 (10% FBS). These results indicate that the model captures FBS-dependent changes in uptake kinetics and extent for BaP, although its broader applicability to other compounds remains to be assessed.

**Figure 2:**
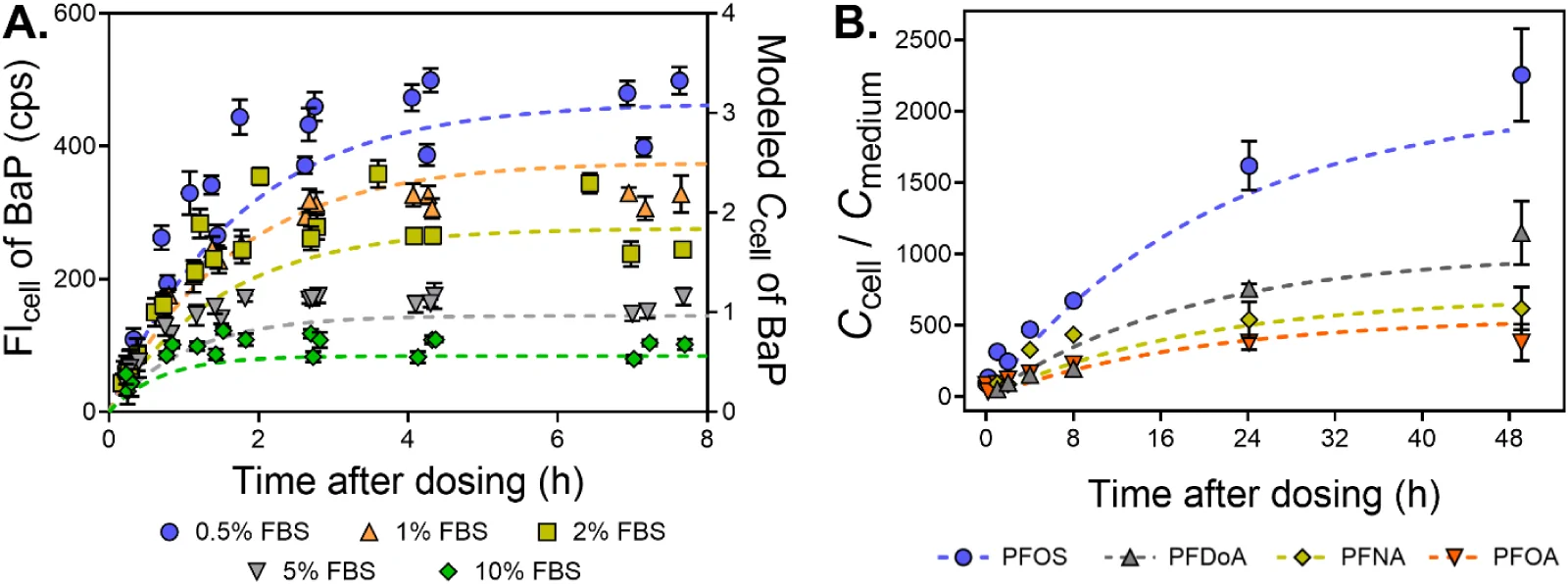
(A) Time-dependent intracellular uptake of benzo­[a]­pyrene (BaP) expressed as relative fluorescence intensity (FI_cell_) in HEK293 cells as a function of increasing fetal bovine serum (FBS) content (0.5–10%). Experiments from Fischer et al.^27^ and simulations with *Model for Neutrals.R*. Cell growth was adjusted based on the FBS content (Table S6). Symbols are experimental FI_cell_ (mean ± SD),^27^ and dashed lines are simulated cellular concentrations. (B) Time-dependent cellular enrichment ratios (*C*_cell_/*C*_medium_) for PFOS, PFDoA, PFNA, and PFOA in HepG2 cells cultured with 0.5% FBS. Symbols show experimental measurements (mean ± SD), and dashed lines are not fits but predictions using the kinetic model (*Model for IOCs.R*). Cell growth was set to 0 as no cell growth was observed throughout the experiment (Figure S2).

### 4.2 Model Evaluation for PFAS

We measured uptake kinetics of four PFAS (PFOS, PFOA, PFNA, and PFDoA, >99.99% ionized at pH 7.4) in HepG2 cells cultured in 6-well plates (300,000 cells/well, DMEM GlutaMAX with 0.5% FBS). Intracellular accumulation of PFAS proceeded much slower than for neutral chemicals and did not reach equilibrium within the typical exposure time of 24 h in in vitro bioassays (Figure 2B). Since no cell growth was observed over the 48-h experiment (Figure S2), the slower intracellular accumulation was not attributable to growth dilution. The kinetic model was consistent with these slower uptake dynamics and compound-specific trends. For PFOS, the model slightly underestimated *t*_95%_ (63 h modeled vs 89 h experimental), whereas for PFOA it overpredicted *t*_95%_ (59 h vs 30 h). For PFNA and PFDoA, simulated *t*_95%_ values (56–59 h) over- and underestimated experimental values (17 and 126 h), respectively, but lay in the same order of magnitude. Model uncertainty likely stems from the use of *P*_app_ values derived from MDCK-LE cells.^26^ Determining *P*_app_ in cell lines commonly used for in vitro toxicity testing, such as HEK293 or HepG2, could improve *t*_95%_ model predictions. Despite deviations in *t*_95%_, cellular enrichment ratios (*C*_cell_/*C*_medium_) after 48 h were predicted within 0.6- to 1.3-fold of measured values for all four PFAS.

### 4.3 Model Evaluation for Volatile Chemicals

Volatilization losses were simulated using a set of 126 hypothetical chemicals with log *K*_ow_ ranging from 1 to 6 and log *K*_aw_ between −10 and 10. Additionally, loss to air was calculated for 19 baseline toxicants that were used in a previous study^17^ to define an empirical volatility threshold at a medium-water partition coefficient (*K*_medium/air_) of log 4 (Table S2). Simulations indicated that compounds with log *K*_medium/air_ > 6 retained over 80% of their initial mass after 24 h in medium supplemented with 10% FBS, whereas the majority of those with log *K*_medium/air_ < 4 showed over 80% depletion from the medium (Figure 3). According to the model predictions, the empirical threshold of log *K*_medium/air_ = 4 reported by us previously^17^ appears at least 1 order of magnitude too low. This empirical “volatility cutoff” was based on chemicals cross contaminating neighboring wells and not by chemical analysis. Moreover, the well plate was not entirely open but closed with a breathable foil that might have slowed down the partitioning to air.

**Figure 3:**
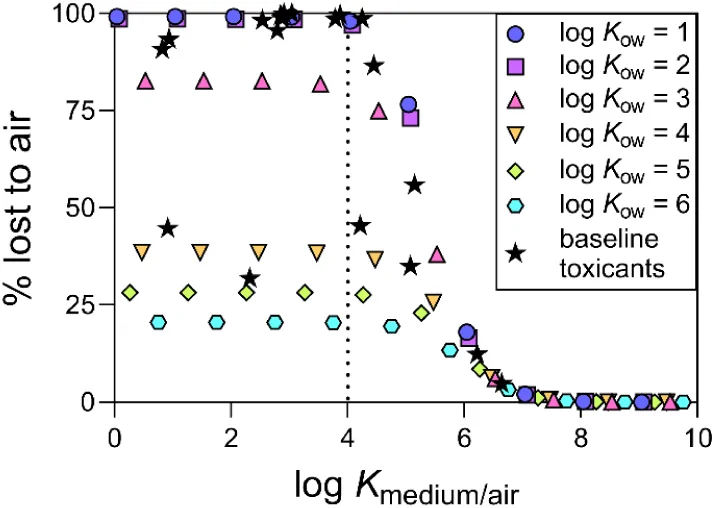
Simulated percent loss to air over 24 h versus medium-air partition coefficients (*K*_medium/air_) for 126 fictional chemicals and 19 baseline toxicants (see Table S2 for chemical properties). Simulations were done for 5,000 HepG2 cells in 384-well plates and medium with 10% FBS. The dashed line shows the empirical volatility cutoff.

According to the new simulations in completely open wells, only 2 baseline toxicants, 2-phenylphenol and 3-nitroaniline, are retained in the medium and can be tested in open well plates. Furthermore, the simulation implies that the loss cannot be reliably assessed using the log *K*_medium/air_ alone, because depending on the hydrophobicity (i.e., log *K*_ow_), the loss after 24 h at a given log *K*_medium/air_ can vary widely. For example, two compounds with the same log *K*_medium/air_ of 4, but log *K*_ow_ of 2 and 6 have predicted losses of 97% and 21% after 24 h. This phenomenon can be explained by the very small free fractions in medium (*f*_free,medium_, ratio of freely dissolved mass in the medium to total mass added to the system) of hydrophobic chemicals that can be lost to the air phase, which slows down this loss process. Simulation of loss to air was also run for 7 days leading to very similar losses to air for chemicals with the same log *K*_medium/air_ (Figure S3). More experimental data will be required to reliably define the volatility threshold, especially analytically quantified loss over time in plates covered with breathable foil or only a loose plastic cover.

### 4.4 Model Evaluation against Experimental Cellular Concentrations and Free Fractions

We compared *C*_cell_/*C*_nom_ ratios simulated by the kinetic model after 24 h to published experimental data for 17 neutral compounds measured in Balb/c 3T3, RTgill-W1, primary rat hepatocytes, and MCF-7 cells. The comparisons between simulation and experimental *C*_cell_/*C*_nom_ ratios are shown in Figure S4.^62–65^ Our kinetically resolved model outperformed (RMSE = 0.56 log_10_ units) predictions from two previously published equilibrium mass balance models (RMSE 1.12 and 1.30 log_10_ units).^18,31^ These evaluation data include the rainbow trout gill line RTgill-W1, which is run at 19 °C. Because parameters at 19 °C were not available, the 37 °C parameters were applied to all four cell lines, which adds uncertainty to the RTgill-W1 comparison but could be resolved in the future with partition and diffusion coefficients measured at 19 °C.

Simulated *f*_free,medium_ after 24 h exposure were also compared with 57 measured *f*_free,medium_ compiled from various literature studies (Figure S5).^5,6,9,31,35,36,62^ Of these, 34 predictions (60%) were within 50% of the measured value, 47 (82%) fell within a 3-fold range, and 56 (98%) were within 10-fold. Model performance was consistent for hydrophilic and moderately hydrophobic chemicals, including caffeine (modeled: 82–96%; experimental: 76–106%) and bisphenol A (15–47% vs 15–22%). Across all compounds, the model explained 57% of variance (R^2^ = 0.57) with an RMSE of 0.49 log_10_ units, indicating robust performance across chemical classes and assay conditions.

### 4.5 Simulations across Chemicals and Assay Setups

Simulations across 113 model compounds (Table S1) covering 7 orders of magnitude in lipophilicity, three plate formats, and various medium compositions were used to address three questions: (1) Under which conditions does serum-mediated passive dosing (SMPD), the buffering of chemical losses by reversible binding to serum proteins and lipids,^15,27^ fail to maintain stable medium concentrations? (2) When losses occur, is our previously developed equilibrium mass balance model (MBM) that only accounts for partitioning to medium constituents and cells^20^ sufficient to predict *C*_free,medium_, or is a kinetic model required to account for time-dependent losses to air and well plate sorption? (3) Under which conditions does *C*_free,cell_ deviate from *C*_free,medium_ due to delayed cellular uptake kinetics and cell growth dilution. This distinction is toxicologically relevant because *C*_free,cell_ is the concentration most directly associated with the intracellular target site.^66^ We quantify this deviation using *t*_95%_ and the ratio of the areas under the *C*_free,cell_ and *C*_free,medium_ time curves over 24 h (AUC_free,cell_/AUC_free,medium_). The 113 model compounds comprise 74 neutral and 39 ionizable compounds. The ionizable compounds include 13 cationic compounds, among them a quaternary ammonium compound, 22 anionic compounds, among them ten perfluoroalkyl acids and several pharmaceutical carboxylic acids, and 4 multiprotic compounds.

Simulations were performed across 96-, 384-, and 1536-well formats under three medium scenarios: (i) DMEM + 10% FBS, representing standard serum-supplemented assay conditions; (ii) DMEM:F12 serum-free medium, a standard serum-free formulation, with protein and lipid concentrations derived from experimental quantification (Table S4), representing a realistic reduced-binding scenario; and (iii) serum-free medium with no assigned protein or lipid content, representing a worst-case scenario with no capacity for SMPD. Scenarios (ii) and (iii) represent the potential range of medium binding capacity under serum-free conditions, as the Lowry protein and sulfo-phosphovanillin lipid assays may respond to medium constituents with no or low sorption affinity for organic chemicals.^20^ Time-resolved distribution profiles for all 113 compounds in 384-well plates for 5000 HepG2 cells exposed to 40 μL of DMEM supplemented with 10% FBS are provided in Figures S6–S10.

To address question (1), we defined *f*_medium_ as the fraction of initial chemical mass retained in the medium after 24 h and evaluated under which conditions SMPD fails to maintain *f*_medium_ above 80%. Under 10% FBS in the 384-well format, SMPD effectively prevented *f*_medium_ from falling below 80% for 90 of 113 compounds (Figure 4A). For these compounds, *C*_free,medium_ can be estimated directly from *C*_nom_ using the medium-water partition coefficient (*K*_medium/w_) alone, without the need for more complex equilibrium mass balance or kinetic modeling.^15^ The 23 compounds for which *f*_medium_ fell below 80% were exclusively driven by volatilization, with air losses ranging from 22% to 93%. Well-plate sorption remained below 10% and cellular uptake accounted for less than 16% of total mass even for the most lipophilic neutrals such as BaP (mass fraction in cells (*f*_cell_) = 14%) and BghiP (*f*_cell_ = 16%). Under serum-free conditions, the number of depleted compounds increased to 39 (Figure 4B). Loss to air again dominated for 27 of these compounds, while cellular accumulation exceeded 20% of total mass for 5 highly lipophilic PAHs, including BaP (*f*_cell_ = 49%) and BghiP (*f*_cell_ = 61%). Medium depletion from cellular uptake was negligible (≤5%) for compounds with log *K*_lip/w_ ≤ 4 regardless of serum level, but dominated for compounds with log *K*_lip/w_ > 4 (Figure S11). Well-plate sorption reached up to 16% but exceeded 5% for only 16 compounds. In the absence of medium binding, 61 of 113 compounds showed *f*_medium_ < 80%, with cellular uptake, volatilization, and well-plate sorption each contributing substantially, and well-plate sorption reaching up to 39% for tris­(3,5-dimethylphenyl)­phosphate (*f*_well_ = 39%) (Figure 4C).

**Figure 4:**
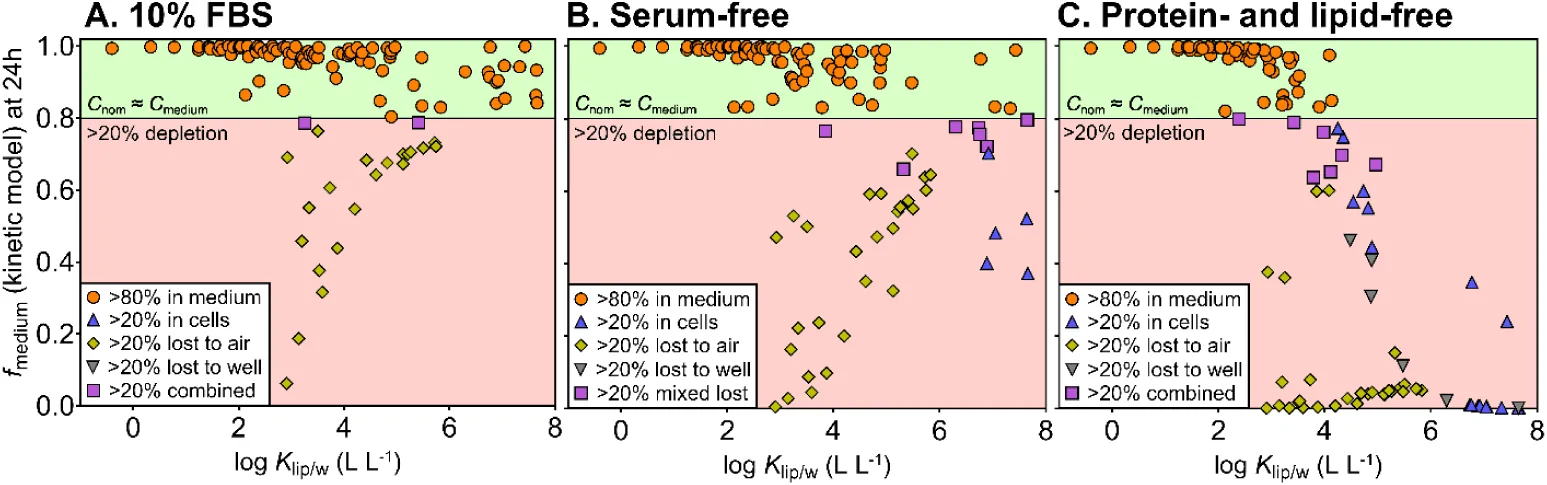
Fraction of initial chemical mass remaining in the medium (*f*_medium_) after 24 h of incubation in a 384-well plate (40 μL medium, 5,000 HepG2 cells) as predicted by the kinetic model, plotted against the phospholipid–water partition coefficient (log *K*_lip/w_) of 113 model chemicals for (A) DMEM supplemented with 10% FBS, (B) serum-free DMEM:F12 with protein and lipid concentrations derived from experimental quantification, and (C) serum-free medium with no assigned protein or lipid content. Symbol shape indicates the dominant loss pathway: compounds retaining ≥80% of initial mass in the medium (green region) can be approximated as *C*_nom_ ≈ C_medium_, while compounds below this threshold (red region) require a distribution model to accurately predict medium concentrations. “>20% combined” indicates that multiple loss pathways collectively exceed 20% depletion.

Depletion increased systematically with plate miniaturization: under 10% FBS, SMPD maintained *f*_medium_ above 80% for 93, 90, and 77 of 113 compounds in the 96-, 384-, and 1536-well formats, respectively (Figures S12–S14). Well-plate sorption increased from 8.3% to 20% across formats, reflecting the higher plastic surface-area-to-medium-volume ratio in smaller wells.^32,67,68^ In the absence of medium binding, *f*_cell_ reached 99% for the most lipophilic compounds in the 1536-well format, demonstrating that miniaturized formats without adequate serum supplementation can result in near-complete medium depletion.

To address question (2), we compared *C*_free,medium_ predictions from the kinetic model to our previously developed equilibrium MBM (Table S11).^20^ At 10% FBS in 384-well plates, both models predicted *C*_free,medium_ within 10% for 83 of 113 compounds, with deviations confined to 30 compounds with >10% volatilization losses (ratios 0.06–0.87, Figure 5A). Under serum-free conditions, agreement decreased (76 of 113 within 10%) as deviations extended beyond volatile compounds. All volatile compounds deviated substantially (ratios 0.005–0.73), while eight additional compounds showed ratios of 0.81–0.88 due to combined air and plastic losses. Five highly lipophilic PAHs with >20% cellular accumulation maintained ratios ≥0.95, but two IOCs, amiodarone and didecyldimethylammonium (DDAC), showed ratios of 3.4 due to overestimation of cellular uptake by the equilibrium model (Figure 5B). Without medium binding, 62 of 113 compounds fell outside 10% agreement. Volatile compounds showed ratios of 0.001–0.82, IOCs with low *P*_app_ reached ratios of 25 (DDAC) and 79 (amiodarone), and ten highly lipophilic neutrals showed ratios of 0.66–0.86 (Figure 5C), because the kinetic model captured concurrent losses to air and plastic that the MBM did not account for. The 10% threshold is a dosimetric tolerance and not a statistical test, as it compares two model predictions. Differences below it fall within the combined analytical and experimental uncertainty of in vitro exposure measurements and do not alter the dosimetric interpretation.

**Figure 5:**
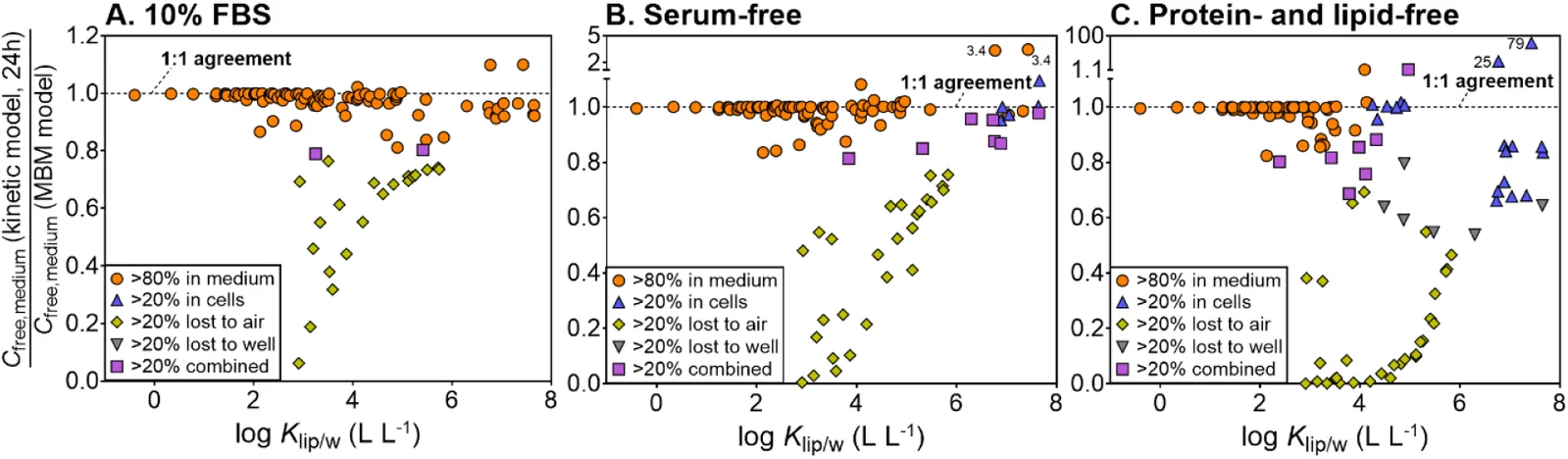
Ratio of *C*_free,medium_ after 24 h of incubation predicted by the kinetic model to *C*_free,medium_ predicted by an equilibrium mass balance model^20^ (MBM) in a 384-well plate (40 μL medium, 5,000 HepG2 cells), plotted against the phospholipid–water partition coefficient (log *K*_lip/w_) of 113 model chemicals for (A) DMEM supplemented with 10% FBS, (B) serum-free DMEM:F12 with protein and lipid concentrations derived from experimental quantification, and (C) serum-free medium with no assigned protein or lipid content. A ratio of 1 (dashed line) indicates perfect agreement between the two models. Ratios below 1 indicate that the MBM may overestimate *C*_free,medium_ due to depletive kinetic processes. Symbol shape indicates the dominant depletion pathway. Values exceeding the lower *y*-axis range are annotated. “>20% combined” indicates that multiple loss pathways collectively exceed 20% depletion.

To address question (3), we tested the impact of delayed uptake kinetics on cellular accumulation by modeling *t*_95%_ in cells for 113 chemicals and compared predicted AUC_free,cell_ to AUC_free,medium_ over 24 h, using a 384-well plate setup (40 μL medium, 5,000 HepG2 cells) under the three medium binding conditions. For most neutral compounds, *t*_95%_ was below 2 h, even for highly lipophilic chemicals such as PCB 180 (*t*_95%_ = 1.6 h). In contrast, several IOCs are expected to exhibit slow uptake, with PFOS reaching *t*_95%_ at 24 h, fexofenadine at 42 h, and DDAC and amiodarone at 44 h (Figure S15). While *P*_app_ has been validated for four PFAS against experimental cellular uptake kinetics (Figure 2B), the applicability of experimental *P*_app_ values for other IOCs remains to be confirmed, particularly where reported *P*_app_ < 1 × 10^–6^. We recommend using the median *P*_app_ of 5 × 10^–6^ cm/s derived from all available IOC data (Figure S16) as a default, when experimental data are not available. This default is derived from 39 IOCs spanning log *K*_ow_ 1.7 to 7.8 (Table S1) and should be treated as an order-of-magnitude estimate.

AUC_free,cell_/AUC_free,medium_ revealed a clear distinction between neutral compounds and certain IOCs. For most neutral compounds, uptake was fast and higher FBS concentrations further accelerated it by enhancing transport across the UWL, the dominant transport barrier for neutral molecules.^69^ At 10% FBS, AUC_free,cell_/AUC_free,medium_ ratios reached 0.92 for BaP and 0.68 for BghiP (Figure S17). Notably, facilitated transport across the UWL may overestimate evaporative loss and cellular uptake rates for very hydrophobic chemicals, while both processes may be underestimated without facilitated transport (Figure S18). Under serum-free conditions, four hydrophobic PAHs showed ratios of 0.40–0.93, and in the absence of medium binding, ratios dropped to 0.13–0.48. These deviations were not caused by slow cellular uptake but by rapid depletion of *C*_free,medium_ to cells, plastic, and air.

For hydrophobic IOCs with low *P*_app_ (<5 × 10^–5^ cm/s), the mechanism was different. AUC_free,cell_/AUC_free,medium_ dropped to 0.35 for PFOS (Figure S17B), reflecting near-constant *C*_free,medium_ maintained by SMPD while *C*_free,cell_ increased gradually beyond 24 h (Figure S19B). Those deviations were consistent across all three medium binding conditions, indicating that discrepancies between AUC_free,cell_ and AUC_free,medium_ for those IOCs are governed by slow membrane permeability rather than medium composition and UWL transport. Higher FBS concentrations did not accelerate uptake but slightly reduced it, as FBS-driven cell proliferation further diluted intracellular concentrations (Figures S17B, S19B). For PFOS, the AUC ratio declined from 0.41 at 0.5% FBS to 0.37 at 10% FBS, consistent with Fischer et al. (2018), where uptake of the permanently anionic 1-pyrene decanoic acid was delayed at higher FBS concentrations at which increased cell growth was observed.^27^

As a practical rule, equilibrium mass balance modeling of *C*_free,medium_ is adequate when losses to volatilization and well-plate sorption stay below 10% of the initial mass, which was demonstrated experimentally in several studies.^9,35,70,71^ Most nonvolatile compounds meet this condition under 10% FBS in 96- and 384-well plates. Kinetic modeling is required when volatilization or sorption exceeds 10%, as under serum-free conditions or in 1536-well plates, or when low membrane permeability prevents cellular uptake from reaching steady state within the assay duration. This second case applies when the modeled *t*_95%_ approaches the exposure time, as it did for several hydrophobic IOCs.

### 4.6 Application of the Parameter Fitting Tool

The parameter fitting tool (*Parameter fit model.R*) was applied to two experimental data sets: BaP in HepG2 and MCF-7 cells^33^ and bisphenol A (BPA) in HepaRG cells.^40^ Volatilization, plastic sorption, UWL transport, membrane permeability, and binding to medium and cells were fixed (Tables S1–S8), leaving CL_met_ as the only unknown parameter. For BaP, 7-ethoxy-resorufin O-deethylation (EROD) activity data were used to fix the observed CYP1A1 induction slope,^33^ leaving the basal clearance rate CL_met_ (0 h) as the fitted parameter. For BPA in HepaRG, a constant CL_met_ was assumed as no induction data was available. Residual variability after fitting was small, with rRMSE of 16%, 29%, and 28% for MCF-7, HepG2, and HepaRG, respectively (Figure 6), indicating that metabolic clearance is the dominant uncharacterized process. These results demonstrate that the model can disentangle intrinsic kinetic rates from competing processes using observed concentration-time profiles in medium and/or cells. The BPA in HepaRG data set originates from the INSIGHT study.^40^ While INSIGHT calibrates partition coefficient corrections and net efflux rates for each cell line, in our model, the disposition parameters were set from first-principles and only CL_met_ was fitted. A direct comparison of prediction accuracy between the two models is therefore not informative, as they rely on different system-specific parameters and serve different purposes.

**Figure 6:**
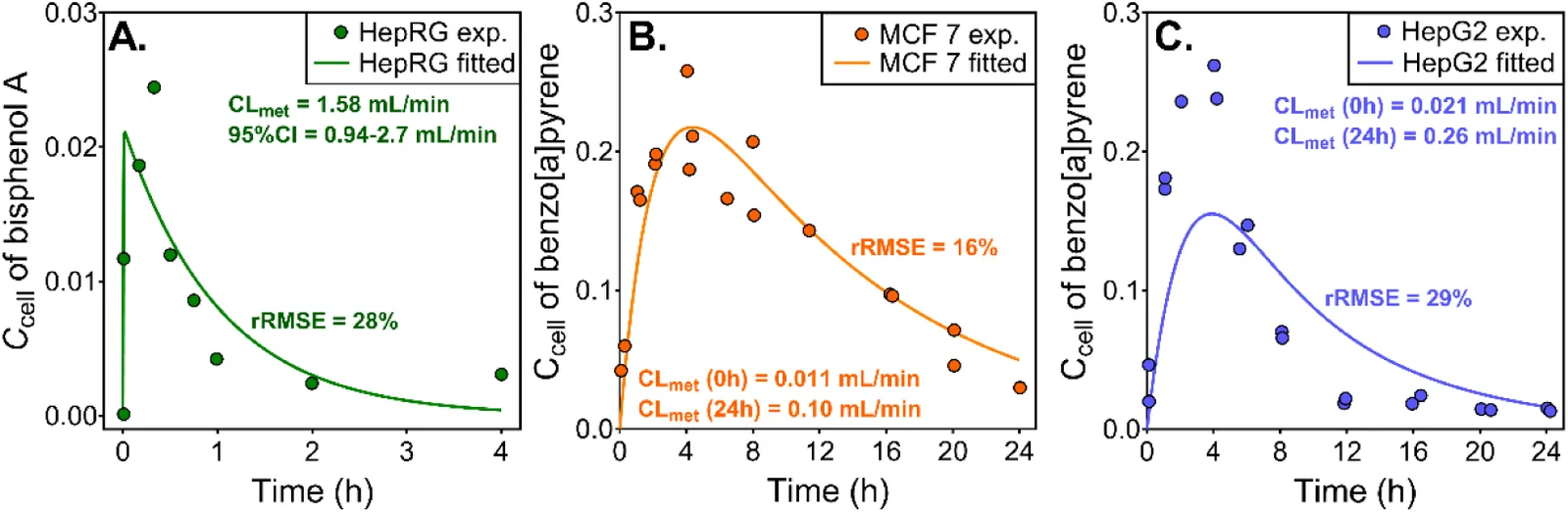
Fitted model predictions and observed total cellular concentrations for (A) bisphenol A in HepaRG cells (12-well plate, 1 mL William’s E + 10% FBS, 800,000 cells/well; Brenner et al. 2026),^40^ (B) benzo­[a]­pyrene in MCF-7 cells (96-well plate, 120 μL DMEM GlutaMAX + 2% FBS, 11,000 cells/well),^33^ and (C) benzo­[a]­pyrene in HepG2 cells (96-well plate, 120 μL DMEM GlutaMAX + 2% d-FBS, 15,000 cells/well).^33^ For benzo­[a]­pyrene, metabolic clearance (CL_met_) increases over the assay duration due to CYP1A1 induction, and initial (0 h) and final (24 h) values are shown. Fluorescence intensities of benzo­[a]­pyrene were scaled to cellular concentrations (Figure 2). Goodness of fit is expressed as relative root mean squared errors (rRMSE, %).

### 4.7 Limitations and Implications for In Vitro Dosimetry and QIVIVE

This study presents the first fully time-resolved mechanistic in vitro disposition model that integrates chemical-specific properties with assay design characteristics to predict time-resolved chemical distribution across air, medium, well plate plastics, and cells under diverse assay conditions. The model reproduced cellular uptake kinetics and captured the differential impact of FBS on neutral and hydrophobic IOCs as observed experimentally and reported previously (Figure 2).^27,62,63^ The model captured losses to volatilization in open well plates (Figure 3), and accurately predicted *f*_free,medium_ across diverse chemicals and assay setups (Figure S5).^5,6,9,31,35,36,62^

Model evaluation against published experimental cellular concentrations was limited to 17 neutral compounds, the compounds for which suitable *C*_cell_ and *C*_nom_ data and model parameters were available. While cellular uptake of anionic compounds was measured for four PFAS (Figure 2B), broader model evaluation will require additional experimental data for diverse IOCs, including carboxylic acids, amines, phenols, and quaternary ammonium compounds.^45^ A further challenge remains superhydrophobic chemicals, where limited experimental data are available (log *K*_ow_ ≥ 7.6 for neutral chemicals), which is not representative for the universe of industrial chemicals.^72^

Several limitations define the applicability domain of the model. Model evaluation remains constrained by limited availability of experimental data on *C*_free,medium_, *C*_cell_, and *P*_app_. For IOCs, no simple structure–activity relationships for *P*_mem_ are available, and existing prediction methods such as COSMOperm are computationally intensive.^69^ The experimental *P*_app_ used as proxies for *P*_mem_ were taken from in vitro permeability models (MDCK, Caco-2, PAMPA) other than the cell lines used in the bioassays, which adds uncertainty to the predicted uptake kinetics and potentially contributed to the *t*_95%_ deviations for PFAS. Measuring *P*_app_ directly in Transwell assays with the cell lines used for standard in vitro toxicity assays would remove this source of uncertainty. A measured *P*_app_ would also capture active transport and lysosomal trapping in that cell line, whereas predicted permeabilities only cover passive transcellular diffusion. Partition and diffusion parameters are fixed at pH of 7.4, even though pH of formulated medium in 5% CO_2_ at 37 °C is rather 7.7 or even higher.^73^ Binding to medium and cells is described using bovine serum albumin, structural protein, and liposomes as proxies for all sorptive phases, which may not fully reflect the compositional complexity of the medium and cells. Where these surrogates prove insufficient, experimental medium-water (*K*_medium/w_) and cell-water (*K*_cell/w_) partition coefficients can be used directly to improve accuracy. Batch-to-batch variability in FBS composition is not represented but can be captured by quantifying the protein and lipid content of the specific serum lot, or by measuring *K*_medium/w_ directly. All of these limitations concern the availability of input parameters rather than the structure of the model, so prediction accuracy is expected to improve as permeability, partitioning, and binding data expand across more chemicals, media and cells used in standard in vitro assays, and assay conditions.

DYNAMEX offers a flexible, mechanistic framework to improve in vitro dosimetry by quantifying how medium composition, assay geometry, and chemical properties influence the stability of *C*_free,medium_ and the kinetics of *C*_free,cell_ establishment. It enables users to define the applicability domain of specific assay setups, for example by flagging volatile compounds unsuitable for open-well systems or identifying chemicals that require time-resolved exposure metrics such as AUC_free,cell_ due to slow cellular uptake. By predicting the full time-course and AUCs of *C*_free,medium_ and *C*_free,cell_, the model provides the exposure metrics that QIVIVE workflows require. It converts the nominal effect concentration of an assay into the freely dissolved or cellular exposure that drives the response, such as AUC_free,cell_ for compounds with slow uptake. This bioeffective concentration replaces *C*_nom_ as the in vitro point of departure. A physiologically based toxicokinetic (PBTK) model then predicts the human external dose that produces the equivalent free concentration in blood or target tissue by reverse dosimetry.^74^ The framework also provides a foundation for extending dosimetry modeling to 3D systems such as spheroids, organoids, and microphysiological platforms.

## Supplementary Material







## Notes

*R* model codes for (1) batch simulations of neutral compounds (*Model for Neutrals.R*), (2) batch simulations of IOCs (*Model for IOCs.R*), and (3) the parameter fitting tool (*Parameter fit model.R*), together with Excel input sheets for system parameters, chemical parameters, and observed data (ZIP)
Chemical fluxes between compartments (Section S1); chemical fluxes in compartments (Section S2); details on Lowry and the sulfo-phosphovanillin (SPV) assays (Section S3); details on cellular uptake kinetics experiments with PFAS (Section S4); additional model evaluation plots (Figures S1–S5); time-resolved chemical distribution plots (Figures S6–S10); predicted chemical mass in cells (Figure S11); chemical fractions across medium, cell, air, and well plate (Figures S12–S14); kinetic plots (Figures S15–S19); references used in the Supporting Information (Section S6) (PDF)
Physicochemical properties of model compounds (Tables S1 and S2); well plate dimensions (Table S3); protein and lipid composition of assay media (Table S4); cell line parameters (Tables S5–S8); measured free fractions used for model evaluation (Table S9); compound parameters and predicted vs experimental *C*_cell_/*C*_nom_ values (Table S10); equilibrium mass balance model predictions (Table S11) (XLSX)

## References

[1] Schmeisser S., Miccoli A., von Bergen M., Berggren E., Braeuning A., Busch W., Desaintes C., Gourmelon A., Grafström R., Harrill J., Hartung T., Herzler M., Kass G. E. N., Kleinstreuer N., Leist M., Luijten M., Marx-Stoelting P., Poetz O., van Ravenzwaay B., Roggeband R., Rogiers V., Roth A., Sanders P., Thomas R. S., Marie Vinggaard A., Vinken M., van de Water B., Luch A., Tralau T. (2023). New approach methodologies in human
regulatory toxicology – Not if, but how and when!. Environ. Int..

[2] Escher, B. ; Neale, P. ; Leusch, F. Bioanalytical Tools in Water Quality Assessment; IWA, 2021. 10.2166/9781789061987

[3] Wetmore B. A. (2015). Quantitative
in vitro-to-in vivo extrapolation in a high-throughput environment. Toxicology.

[4] Henneberger L., Huchthausen J., Wojtysiak N., Escher B. I. (2021). Quantitative In
Vitro-to-In Vivo Extrapolation: Nominal versus Freely Dissolved Concentration. Chem. Res. Toxicol..

[5] Henneberger L., Muhlenbrink M., Heinrich D. J., Teixeira A., Nicol B., Escher B. I. (2020). Experimental
Validation of Mass Balance Models for
in Vitro Cell-Based Bioassays. Environ. Sci.
Technol..

[6] Henneberger L., Huchthausen J., König M., Menge A., Wojtysiak N., Escher B. I. (2022). Experimental exposure assessment of designed chemical
mixtures in cell-based in vitro bioassays. Front.
Environ. Chem..

[7] Qin W., Henneberger L., Glüge J., König M., Escher B. I. (2024). Baseline Toxicity
Model to Identify the Specific and
Nonspecific Effects of Per- and Polyfluoroalkyl Substances in Cell-Based
Bioassays. Environ. Sci. Technol..

[8] Nicol B., Vandenbossche-Goddard E., Thorpe C., Newman R., Patel H., Yates D. (2024). A workflow to practically apply true
dose considerations to in vitro testing for next generation risk assessment. Toxicology.

[9] Huchthausen J., König M., Escher B. I., Henneberger L. (2023). Experimental
exposure assessment for in vitro cell-based bioassays in 96- and 384-well
plates. Front. Toxicol..

[10] Henneberger L., Klüver N., Mühlenbrink M., Escher B. (2022). Trout and Human Plasma
Protein Binding of Selected Pharmaceuticals Informs the Fish Plasma
Model. Environ. Toxicol. Chem..

[11] Qin W., Escher B. I., Huchthausen J., Fu Q., Henneberger L. (2024). Species Difference?
Bovine, Trout, and Human Plasma Protein Binding of Per- and Polyfluoroalkyl
Substances. Environ. Sci. Technol..

[12] Huang R., Sakamuru S., Martin M. T., Reif D. M., Judson R. S., Houck K. A., Casey W., Hsieh J.-H., Shockley K. R., Ceger P., Fostel J., Witt K. L., Tong W., Rotroff D. M., Zhao T., Shinn P., Simeonov A., Dix D. J., Austin C. P., Kavlock R. J., Tice R. R., Xia M. (2014). Profiling of the Tox21 10K compound library for agonists and antagonists
of the estrogen receptor alpha signaling pathway. Sci. Rep..

[13] Attene-Ramos M. S., Miller N., Huang R., Michael S., Itkin M., Kavlock R. J., Austin C. P., Shinn P., Simeonov A., Tice R. R., Xia M. (2013). The Tox21 robotic platform for the
assessment of environmental chemicals - from vision to reality. Drug Discovery Today.

[14] Hsieh J. -H., Huang R. L., Lin J. -A., Sedykh A., Zhao J., Tice R. R., Paules R. S., Xia M., Auerbach S. S. (2017). Real-time
cell toxicity profiling of Tox21 10K compounds reveals cytotoxicity
dependent toxicity pathway linkage. PLoS One.

[15] Fischer F. C., Henneberger L., Schlichting R., Escher B. I. (2019). How To Improve the
Dosing of Chemicals in High-Throughput in Vitro Mammalian Cell Assays. Chem. Res. Toxicol..

[16] Escher B. I., Henneberger L., König M., Schlichting R., Fischer F. C. (2020). Cytotoxicity Burst?
Differentiating Specific from Nonspecific
Effects in Tox21 in Vitro Reporter Gene Assays. Environ. Health Perspect.

[17] Escher B. I., Glauch L., König M., Mayer P., Schlichting R. (2019). Baseline Toxicity
and Volatility Cutoff in Reporter Gene Assays Used for High-Throughput
Screening. Chem. Res. Toxicol..

[18] Armitage J. M., Wania F., Arnot J. A. (2014). Application of Mass Balance Models
and the Chemical Activity Concept To Facilitate the Use of in Vitro
Toxicity Data for Risk Assessment. Environ.
Sci. Technol..

[19] Proença S., Escher B. I., Fischer F. C., Fisher C., Grégoire S., Hewitt N. J., Nicol B., Paini A., Kramer N. I. (2021). Effective
exposure of chemicals in in vitro cell systems: A review of chemical
distribution models. Toxicol. In Vitro.

[20] Fischer F. C., Henneberger L., König M., Bittermann K., Linden L., Goss K.-U., Escher B. I. (2017). Modeling Exposure
in the Tox21 in Vitro Bioassays. Chem. Res.
Toxicol..

[21] Fisher C., Siméon S., Jamei M., Gardner I., Bois Y. F. V. (2019). Virtual
in vitro distribution model for the mechanistic prediction of intracellular
concentrations of chemicals in in vitro toxicity assays. Toxicol. In Vitro.

[22] Armitage J. M., Sangion A., Parmar R., Looky A. B., Arnot J. A. (2021). Update
and Evaluation of a High-Throughput In Vitro Mass Balance Distribution
Model: IV-MBM EQP v2.0. Toxics.

[23] Groothuis F. A., Heringa M. B., Nicol B., Hermens J. L. M., Blaauboer B. J., Kramer N. I. (2015). Dose metric considerations
in in vitro assays to improve
quantitative in vitro-in vivo dose extrapolations. Toxicology.

[24] Bittermann K., Goss K. U. (2017). Predicting apparent passive permeability
of Caco-2
and MDCK cell-monolayers: A mechanistic model. PLoS One.

[25] Goetz G. H., Shalaeva M., Caron G., Ermondi G., Philippe L. (2017). Relationship
between Passive Permeability and Molecular Polarity Using Block Relevance
Analysis. Mol. Pharmaceutics.

[26] Ryu S., Yamaguchi E., Sadegh Modaresi S. M., Agudelo J., Costales C., West M. A., Fischer F., Slitt A. L. (2024). Evaluation of 14
PFAS for Permeability and Organic Anion Transporter Interactions:
Implications for Renal Clearance in Humans. Chemosphere.

[27] Fischer F. C., Abele C., Droge S. T. J., Henneberger L., König M., Schlichting R., Scholz S., Escher B. I. (2018). Cellular
Uptake Kinetics of Neutral and Charged Chemicals in in Vitro Assays
Measured by Fluorescence Microscopy. Chem. Res.
Toxicol..

[28] Gulden M., Morchel S., Tahan S., Seibert H. (2002). Impact of protein binding
on the availability and cytotoxic potency of organochlorine pesticides
and chlorophenols in vitro. Toxicology.

[29] Gulden M., Seibert H. (1997). Influence of protein binding and
lipophilicity on the
distribution of chemical compounds in in vitro systems. Toxicol. In Vitro.

[30] Seibert H., Morchel S., Gulden M. (2002). Factors influencing
nominal effective
concentrations of chemical compounds in vitro: medium protein concentration. Toxicol. In Vitro.

[31] Kramer N. I., Krismartina M., Rico-Rico A., Blaauboer B. J., Hermens J. L. M. (2012). Quantifying Processes
Determining the Free Concentration
of Phenanthrene in Basal Cytotoxicity Assays. Chem. Res. Toxicol..

[32] Fischer F. C., Cirpka O. A., Goss K. U., Henneberger L., Escher B. I. (2018). Application of Experimental Polystyrene Partition Constants
and Diffusion Coefficients to Predict the Sorption of Neutral Organic
Chemicals to Multiwell Plates in in Vivo and in Vitro Bioassays. Environ. Sci. Technol..

[33] Fischer F. C., Abele C., Henneberger L., Kluver N., Konig M., Muhlenbrink M., Schlichting R., Escher B. I. (2020). Cellular Metabolism
in High-Throughput In Vitro Reporter Gene Assays and Implications
for the Quantitative In Vitro–In Vivo Extrapolation. Chem. Res. Toxicol..

[34] Huchthausen J., Henneberger L., Mälzer S., Nicol B., Sparham C., Escher B. I. (2022). High-Throughput
Assessment of the Abiotic Stability
of Test Chemicals in In Vitro Bioassays. Chem.
Res. Toxicol..

[35] Henneberger L., Muhlenbrink M., Konig M., Schlichting R., Fischer F. C., Escher B. I. (2019). Quantification
of freely dissolved
effect concentrations in in vitro cell-based bioassays. Arch. Toxicol..

[36] Birch H., Kramer N. I., Mayer P. (2019). Time-Resolved
Freely Dissolved Concentrations
of Semivolatile and Hydrophobic Test Chemicals in In Vitro Assays—Measuring
High Losses and Crossover by Headspace Solid-Phase Microextraction. Chem. Res. Toxicol..

[37] Caneparo C., Chabaud S., Fradette J., Bolduc S. (2022). Evaluation of a Serum-Free
Medium for Human Epithelial and Stromal Cell Culture. Int. J. Mol. Sci..

[38] van
der Valk J., Brunner D., De Smet K., Fex Svenningsen Å., Honegger P., Knudsen L. E., Lindl T., Noraberg J., Price A., Scarino M. L., Gstraunthaler G. (2010). Optimization
of chemically defined cell culture media – Replacing fetal
bovine serum in mammalian in vitro methods. Toxicol. In Vitro.

[39] Bloch S., Arnot J. A., Kramer N. I., Armitage J. M., Verner M.-A. (2022). Dynamic
Mass Balance Modeling for Chemical Distribution Over Time in In Vitro
Systems With Repeated Dosing. Front. Toxicol..

[40] Brenner D., Bernal K., Sychrová E., Kohoutek J., Langouet S., Tebby C., Beaudouin R., Boufalaas R., Dairou J., Tête A. (2026). Chemical fate in vitro:
A physiological biokinetic (PBK) model for cell-based assays. Altex.

[41] Summerfield S. G., Yates J. W. T., Fairman D. A. (2022). Free Drug
Theory - No Longer Just
a Hypothesis?. Pharm. Res..

[42] Ter
Laak T. L., Van Eijkeren J. C. H., Busser F. J. M., Van
Leeuwen H. P., Hermens J. L. M. (2009). Facilitated Transport of Polychlorinated
Biphenyls and Polybrominated Diphenyl Ethers by Dissolved Organic
Matter. Environ. Sci. Technol..

[43] Ter
Laak T. L., Ter Bekke M. A., Hermens J. L. M. (2009). Dissolved Organic
Matter Enhances Transport of PAHs to Aquatic Organisms. Environ. Sci. Technol..

[44] DelRaso N. J., Foy B. D., Gearhart J. M., Frazier J. M. (2003). Cadmium Uptake Kinetics
in Rat Hepatocytes: Correction for Albumin Binding. Toxicol. Sci..

[45] Escher B. I., Abagyan R., Embry M., Klüver N., Redman A. D., Zarfl C., Parkerton T. F. (2019). Recommendations
for Improving Methods and Models for Aquatic Hazard Assessment of
Ionizable Organic Chemicals. Environ. Toxicol.
Chem..

[46] Seidensticker S., Grathwohl P., Lamprecht J., Zarfl C. (2018). A combined experimental
and modeling study to evaluate pH-dependent sorption of polar and
non-polar compounds to polyethylene and polystyrene microplastics. Environ. Sci. Eur..

[47] Schwarzenbach, R. P. ; Gschwend, P. M. ; Imboden, D. M. Environmental Organic Chemistry, third ed.; Wiley, 2016.

[48] Soetaert K., Petzoldt T., Setzer R. W. (2010). Solving Differential Equations in
R: Package deSolve. J. Stat. Software.

[49] Henneberger L., Mühlenbrink M., Fischer F. C., Escher B. I. (2019). C18-Coated Solid-Phase
Microextraction Fibers for the Quantification of Partitioning of Organic
Acids to Proteins, Lipids, and Cells. Chem.
Res. Toxicol..

[50] Henneberger L., Goss K.-U., Endo S. (2016). Partitioning
of Organic Ions to Muscle
Protein: Experimental Data, Modeling, and Implications for in Vivo
Distribution of Organic Ions. Environ. Sci.
Technol..

[51] Henneberger L., Goss K.-U., Endo S. (2016). Equilibrium Sorption of Structurally
Diverse Organic Ions to Bovine Serum Albumin. Environ. Sci. Technol..

[52] Endo S., Goss K. -U. (2011). Serum Albumin Binding
of Structurally Diverse Neutral
Organic Compounds: Data and Models. Chem. Res.
Toxicol..

[53] Endo S., Escher B. I., Goss K. U. (2011). Capacities
of Membrane Lipids to
Accumulate Neutral Organic Chemicals. Environ.
Sci. Technol..

[54] Droge S. T. J. (2019). Membrane-Water
Partition Coefficients to Aid Risk Assessment of Perfluoroalkyl Anions
and Alkyl Sulfates. Environ. Sci. Technol..

[55] Ulrich, N. ; Endo, S. ; Brown, T. N. ; Watanabe, N. ; Bronner, G. ; Abraham, M. H. ; Goss, K. U. UFZ-LSER database v 3.2; Helmholtz Centre for Environmental Research-UFZ: Leipzig, Germany., 2017. https://www.ufz.de/lserd/

[56] Krause S., Ulrich N., Goss K. U. (2018). Desorption
kinetics of organic chemicals
from albumin. Arch. Toxicol..

[57] Hayduk W., Laudie H. (1974). Prediction of diffusion
coefficients for nonelectrolytes
in dilute aqueous solutions. AIChE J..

[58] Chary S. R., Jain R. K. (1989). Direct measurement of interstitial convection and diffusion
of albumin in normal and neoplastic tissues by fluorescence photobleaching. Proc. Natl. Acad. Sci. U. S. A..

[59] Corning product selection guide; 2026. https://www.corning.com/catalog/cls/documents/drawings/MicroplateDimensions96-384-1536.pdf.

[60] Warne D. J., Baker R. E., Simpson M. J. (2017). Optimal
Quantification of Contact
Inhibition in Cell Populations. Biophys. J..

[61] Areiza J. A., Becanova J., Vojta S., Ganglbauer J., Apte U., Brunetti S. K. L., Haque F., Kim E., Sunderland E. M., Lohmann R., Fischer F. C., Slitt A. (2026). Presence of
Legacy and Emerging PFAS in Human Livers Specimens in the United States
from 2000–2024. Environ. Sci. Technol..

[62] Dimitrijevic D., Fabian E., Nicol B., Funk-Weyer D., Landsiedel R. (2022). Toward Realistic Dosimetry In Vitro:
Determining Effective
Concentrations of Test Substances in Cell Culture and Their Prediction
by an In Silico Mass Balance Model. Chem. Res.
Toxicol..

[63] Scherer M. N., Feshuk M., Armitage J. M., Arnot J. A., Crizer D., DeVito M., Ferguson S. S., Freeman K., Honda G. S., Harrill J. A., Sangion A., Tebes-Stevens C., Paul Friedman K., Wambaugh J. F. (2026). Characterizing Accuracy
of Model
Predictions for Chemical Concentration in High Throughput Screening
Assays. Environ. Sci. Technol..

[64] Stadnicka-Michalak J., Tanneberger K., Schirmer K., Ashauer R. (2014). Measured and Modeled
Toxicokinetics in Cultured Fish Cells and Application to In Vitro
- In Vivo Toxicity Extrapolation. PLoS One.

[65] Truisi G. L., Consiglio E. D., Parmentier C., Savary C. C., Pomponio G., Bois F., Lauer B., Jossé R., Hewitt P. G., Mueller S. O., Richert L., Guillouzo A., Testai E. (2015). Understanding the biokinetics
of ibuprofen after single
and repeated treatments in rat and human in vitro liver cell systems. Toxicol. Lett..

[66] Escher B. I., Hermens J. L. M. (2004). Internal exposure:
Linking bioavailability to effects. Environ.
Sci. Technol..

[67] Schreiber R., Altenburger R., Paschke A., Kuster E. (2008). How to deal with lipophilic
and volatile organic substances in microtiter plate assays. Environ. Toxicol. Chem..

[68] Riedl J., Altenburger R. (2007). Physicochemical substance properties as indicators
for unreliable exposure in microplate-based bioassays. Chemosphere.

[69] Schwöbel J. A.
H., Ebert A., Bittermann K., Huniar U., Goss K.-U., Klamt A. (2020). COSMOperm:
Mechanistic Prediction of Passive Membrane Permeability
for Neutral Compounds and Ions and Its pH Dependence. J. Phys. Chem. B.

[70] Huchthausen J., Mühlenbrink M., König M., Escher B. I., Henneberger L. (2020). Experimental
Exposure Assessment of Ionizable Organic Chemicals in In Vitro Cell-Based
Bioassays. Chem. Res. Toxicol..

[71] Henneberger L., Bardehly S., König M., Escher B. I. (2025). Gaining confidence
in in vitro toxicity data with measured exposure concentrations. NAM J..

[72] Escher B. I. (2026). “The
Dose Makes the Poison”: Relevance of Paracelsus’s Principle
for Modern Chemical Hazard Assessment with New Approach Methodologies. Environ. Sci. Technol..

[73] Michl J., Park K. C., Swietach P. (2019). Evidence-based
guidelines for controlling
pH in mammalian live-cell culture systems. Commun.
Biol..

[74] Fischer F. C., Thackray C., Ferguson N., Chicoine C., Skende O., Hu Z., Zhu Y., Slitt A., Sunderland E. M. (2025). Understanding
Mechanisms of PFAS Absorption, Distribution, and Elimination Using
a Physiologically Based Toxicokinetic Model. Environ. Sci. Technol..

