# Supplementary material for: DYNAMEX: A Time-Resolved Model for Predicting Distribution of Chemicals in In Vitro Cell Assays
Source: Environ Sci Technol. 2026 Aug 26;60(35):24617–29. doi: 10.1021/acs.est.6c06087 (PMC13564414; doi:10.1021/acs.est.6c06087)
Supplement: Supplementary file 1 [file es6c06087_si_002.pdf]

Supporting Information

DYNAMEX: A time-resolved model for predicting distribution of chemicals in in-vitro cell assays

Fabian C. Fischer[1,2,3,], Liam Geyer[2], Miguel Rodriguez[2], Luise Henneberger[3], Beate I. Escher[3,4]*

[1] Department of Pathology and Laboratory Medicine, Brown University, Providence, RI 02912, United States

[2] Department of Biomedical and Pharmaceutical Sciences, University of Rhode Island, Kingston, RI 02881, United States.

[3] Helmholtz Centre for Environmental Research - UFZ, Department Cell Toxicology, Permoserstraße 15, 04318 Leipzig, Germany

[4] Eberhard Karls University Tübingen, Department of Geosciences, 72074 Tübingen, Germany

***Address correspondence to:** fabian_fischer@brown.edu

Section S-1. Chemical Fluxes between Compartments.

Flux from Medium to Cells. The compound flux from the aqueous fraction of the medium to the cells ($J_{\text{free,medium}\rightarrow\text{free,cell}}$, mol/s) is described by a first-order permeability equation:

$$J_{\text{free,medium}\rightarrow\text{free, cell}} = P_{\text{app}} \cdot A_{\text{cell}} \cdot \left(C_{\text{free,medium}} - C_{\text{free,cell}}\right) \quad \text{(S1)}$$

A_{cell} is the surface area of the cells that is in direct contact with the medium (cm²). We assumed that cell growth occurs by proliferation linearly with cell volume::

$$A_{\text{cell}} = A_{\text{cell,0}} \cdot \left(\frac{V_{\text{cell}}}{V_{\text{cell,0}}}\right) \quad \text{(S2)}$$

$A_{\text{cell,0}}$ and $V_{\text{cell,0}}$ are the initial surface area and volume of the cells at time 0.

The apparent permeability coefficient (P_{app}, cm/s) represents overall resistance to transport from the medium into the cytosol of the cell, accounting for both the unstirred water layer (UWL) and cell membrane diffusion. P_{app} is calculated from two sequential transport resistances: diffusion across the UWL (R_{UWL}) and diffusion across the membrane (R_{mem}), as:

$$P_{\text{app}} = \frac{1}{R_{\text{UWL}} + R_{\text{mem}}} \quad \text{(S3)}$$

For neutral chemicals, R_{mem} were derived from the thickness of the cell membrane (x_{mem}, cm), the diffusion coefficient of the chemical in the membrane (D_{mem}, cm²/s), and its membrane-water partition coefficient ($K_{\text{mem/w}}$, $\text{mL}_{\text{w}}/\text{mL}_{\text{mem}}$):

$$R_{\text{mem}} = \frac{x_{mem}}{D_{\text{mem}} \cdot K_{\text{mem/w}}} \quad \text{(S4)}$$

For IOCs, R_{mem} was estimated from literature-reported membrane permeabilities (P_{mem}, cm/s) determined in cell monolayer assays. Because monolayer permeability reflects transport across two membranes (apical and basolateral) plus intracellular diffusion in the cytosol, the resistance for uptake across a single cell membrane was calculated as $R_{\text{mem}} = (1/P_{\text{mem}})/2$, neglecting the intracellular resistance.

R_{UWL} for both neutral and ionizable compounds depends on the UWL thickness (x_{UWL}, cm) and the diffusion coefficient of the chemical in water. To account for facilitated transport of protein- and lipid-bound chemicals across the UWL,[1,2] we incorporated the diffusion coefficients of proteins ($D_{\text{prot,w}}$, cm²/s) in water, the volume fraction of medium proteins

($VF_{\text{prot,medium}}$), and the medium protein-water partition coefficient ($K_{\text{prot/w}}$):

$$R_{\text{UWL}} = \frac{x_{\text{UWL}}}{D_{\text{w}} + D_{\text{prot}} \cdot VF_{\text{prot,medium}} \cdot K_{\text{prot/w}}} \quad \text{(S5)}$$

Flux from Medium to Well Plate. The compound flux from the aqueous fraction of the medium to the well plate ($J_{\text{free,medium}\to\text{well}}$, mol/s) was described by a modified mixed-layer equation:

$$J_{\text{free,medium}\to\text{well}} = \frac{0.5 \cdot A_{\text{well}} \cdot D_{\text{well}} \cdot K_{\text{well/w}}}{dx_{\text{well}}} \cdot \left(C_{\text{free,medium}} - \frac{C_{\text{well}}[1]}{K_{\text{well/w}}}\right) \quad \text{(S6)}$$

$C_{\text{well}}[1]$ is the concentration in the first layer of the well at the medium-well interface (mol/mL$_{\text{well}}$). Transport resistance is governed by the surface area of the well plate in contact with the medium (A_{well}, cm²), the thickness of the outer well layer (dx_{well}, cm), the diffusion coefficient in the well (D_{well}, cm²/s), and the well-water partition coefficient ($K_{\text{well/w}}$, mL$_{\text{w}}$/mL$_{\text{well}}$) that describes the equilibrium to be established at the medium-well interface, assuming that only freely dissolved chemicals can diffuse into the plastic. While the model assumes the medium is well-mixed (i.e., no diffusion limitation on the aqueous side), this simplification ignores the presence of an UWL adjacent to the plastic surface. However, because diffusion in plastic is orders of magnitude slower than in water ($D_{\text{well}} \approx 1\times10^{-12}$ cm²/s vs. $D_{\text{w}} \approx 1\times10^{-6}$ cm²/s),[3] the contribution of the UWL to the overall resistance is negligible.

Flux from Medium to Headspace*.* Volatilization from the medium to the headspace in an "open" multi-well plate setup covered with air-permeable plate sealants and/or plastic lid is described assuming an effectively infinite air volume, such that the air-side concentration does not significantly affect the flux. The compound flux from the freely dissolved fraction in the medium to the air ($J_{\text{free,medium}\to\text{air}}$, mol/s) is given by a mixed-layer equation:

$$J_{\text{free,medium}\to\text{air}} = \frac{A_{\text{medium/air}}}{\frac{x_{\text{medium}}}{D_{\text{w}}} + \frac{1}{K_{\text{aw}}}} \cdot C_{\text{free,medium}} \quad \text{(S7)}$$

$A_{\text{medium/air}}$ is the surface area of the medium in contact with the head space (cm²), x_{medium} is the height of the medium in the well plate (cm), and K_{aw} is the air-water partition coefficient (mL$_{\text{w}}$/mL$_{\text{air}}$). x_{medium} (cm) was calculated as V_{medium} / A_{well}, where V_{medium} is the nominal medium volume and A_{well} is the well bottom surface area, assuming flat-bottom, straight-walled wells.

The expression reflects a one-sided boundary condition, in which the driving force for volatilization is the concentration in the aqueous phase. The air-side resistance is included through $1/K_{aw}$, but the air is assumed to be well-ventilated or of sufficient volume to prevent back diffusion. Cross-contamination of volatile chemicals between neighboring wells has been reported in multi-well plate experiments.[4] Even commonly used plate sealants are not airtight, making open-plate formats unsuitable for volatile compounds. Although not ideal for testing volatile chemicals, this flux term was included in the model to identify when volatilization becomes significant. In such cases, experiments should be conducted in closed systems to avoid uncontrolled loss and cross-well contamination.

Section S2. Chemical Flux in Compartments.

Chemical Flux in Medium. The bioassay medium is considered a homogeneous, well-mixed aqueous suspension with suspended proteins and lipids contained in the base medium and FBS. At the start of the experiment, chemicals are assumed to be in equilibrium with proteins and lipids in the medium. This is achieved by pre-equilibrating the chemical in bioassay medium for 1-24 h prior to dosing.[5] Direct spiking of DMSO stocks into cell plates, *e.g.*, via nanoliter pin devices, bypasses this equilibration step and introduces additional kinetic uncertainties.[6] The present model should therefore only be applied to pre-equilibrated medium solutions.

The chemical flux between proteins and lipids and the aqueous phase in the medium ($J_{\text{bound,medium}\rightarrow\text{free,medium}}$, mol/s) is calculated assuming first-order kinetics, where k_{des} is the desorption rate constant (1/s) and the product $k_{\text{des}} \cdot K_{\text{prot+lip,medium/w}}$ is the effective absorption rate coefficient, where $K_{\text{prot+lip,medium/w}}$ is the medium-water partition coefficient ($\text{mL}_{\text{w}}/\text{mL}_{\text{prot+lip,medium}}$).

$$J_{\text{bound,medium}\rightarrow\text{free,medium}} = k_{\text{des}} \cdot V_{\text{prot+lip,medium}} \cdot \left(C_{\text{bound,medium}} - C_{\text{free,medium}} \cdot K_{\text{prot+lip,medium/w}}\right) \tag{S8}$$

$K_{\text{prot+lip,medium/w}}$ is calculated from the volume fractions of proteins and lipids and the chemical's

binding affinity to these medium constituents:

$$K_{\mathrm{prot+lip,medium/w}} = VF_{\mathrm{prot,medium}} \cdot K_{\mathrm{prot/w}} + VF_{\mathrm{lip,medium}} \cdot K_{\mathrm{lip/w}} \qquad \text{(S9)}$$

$VF_{\mathrm{prot,medium}}$ and $VF_{\mathrm{lip,medium}}$ are the volume fractions of proteins and lipids in the medium, with $VF_{\mathrm{prot,medium}} + VF_{\mathrm{lip,medium}} = 1$, and $K_{\mathrm{prot/w}}$ and $K_{\mathrm{lip/w}}$ are the protein-water and lipid-water partition coefficients of the test chemical ($\mathrm{mL_w/mL_{prot}}$, $\mathrm{mL_w/mL_{lip}}$).

The chemical flux due to abiotic degradation in the medium ($J_{\mathrm{free,medium}\rightarrow\mathrm{AD}}$, mol/s) is calculated with a first-order equation, assuming that only $C_{\mathrm{free,medium}}$ can be degraded:

$$J_{\mathrm{free,medium}\rightarrow\mathrm{AD}} = \mathrm{CL_{abiot}} \cdot C_{\mathrm{free,medium}} \qquad \text{(S10)}$$

$\mathrm{CL_{abiot}}$ is the abiotic clearance rate ($\mathrm{mL_{w,medium}}$/s).

Chemical Flux in Cells. The cell cytoplasm is considered a homogeneous, well-mixed aqueous suspension of cell proteins, while the cell membrane is assumed to be a homogeneous phase of phospholipid bilayers. Although intrinsic membrane permeability is explicitly modeled (eq. S3), concentration gradients within the membrane are not resolved. Instead, as for protein-bound fractions, desorption from membrane lipids is modeled using first-order kinetics. The chemical flux between sorptive phases (membrane and cell proteins) and the aqueous phase in the cells ($J_{\mathrm{bound,cell}\rightarrow\mathrm{free,cell}}$, mol/s) is calculated as:

$$J_{\mathrm{bound,cell}\rightarrow\mathrm{free,cell}} = k_{\mathrm{des}} \cdot V_{\mathrm{prot+lip,cell}} \cdot \left(C_{\mathrm{bound,cell}} - C_{\mathrm{free,cell}} \cdot K_{\mathrm{prot+lip,cell/w}}\right) \qquad \text{(S11)}$$

$V_{\mathrm{prot+lip,cell}}$ is the sum of volume of sorptive proteins and lipids in the cells ($\mathrm{mL_{prot+lip}}$), $K_{\mathrm{prot+lip,cell/w}}$ is the cell protein and lipid-water partition coefficient ($\mathrm{mL_w/mL_{prot+lip}}$), and $C_{\mathrm{bound,cell}}$ is the concentration of chemicals bound to proteins and lipids in the cells ($\mathrm{mol/mL_{prot+lip}}$). $K_{\mathrm{prot+lip,cell/w}}$ is derived from the protein and lipid volume fractions and associated partition coefficients:

$$K_{\mathrm{prot+lip,cell/w}} = VF_{\mathrm{prot,cell}} \cdot K_{\mathrm{prot/w}} + VF_{\mathrm{lip,cell}} \cdot K_{\mathrm{lip/w}} \qquad \text{(S12)}$$

$VF_{\mathrm{prot,cell}}$ and $VF_{\mathrm{lip,cell}}$ are the volume fractions of proteins and lipids in the cells, with $VF_{\mathrm{prot,cell}} + VF_{\mathrm{lip,cell}} = 1$.

The chemical concentration in the cell membrane (C_{mem}, $\mathrm{mol/mL_{lipid}}$) is calculated assuming equilibrium between $C_{\mathrm{free,cell}}$ and membrane concentration according to $K_{\mathrm{mem/w}}$:

$$C_{\mathrm{mem}} = C_{\mathrm{free,cell}} \cdot K_{\mathrm{mem/w}} \qquad \text{(S13)}$$

The chemical flux due to metabolism in the cell ($J_{\text{free,cell}\rightarrow\text{met}}$, mol/s) is calculated with a first-order metabolism equation, assuming that only $C_{\text{free,cell}}$ can be metabolized:

$$J_{\text{free,cell}\rightarrow\text{met}} = \text{CL}_{\text{met}} \cdot C_{\text{free,cell}} \quad \text{(S14)}$$

CL_{met} is the metabolic clearance rate ($\text{mL}_{\text{w,cell}}$/s), which is effectively an additional elimination process as metabolites formed are not considered in the model.

Chemical Flux in Well Plate. Loss to the plastic plates is a two-step process, with an initial fast partitioning and then slow diffusion into the plastic material of the multi-well plate. The temporal change in concentration in the outer well plate layer that is in contact with the medium ($dC_{\text{well}}[1]/dt$, mol/mL_{well}/s) depends on the chemical flux from the medium ($J_{\text{free,medium}\rightarrow\text{well}}$, eq. S6):

$$\frac{dC_{\text{well}}[1]}{dt} = \frac{J_{\text{free,medium}\rightarrow\text{well}}}{dx_{\text{well}} \cdot A_{\text{well}}} \quad \text{(S15)}$$

A_{well} is the surface area of the well plate (cm²), dx_{well} is the thickness of the outer well layer (cm).

To model the diffusion inside the well plastic, we employed the finite difference method to approximate the change in concentration for each layer over time. We used the general diffusion equation describing the second spatial derivative of the concentration (*d²C/dx²)*:

$$\frac{dC}{\text{dt}} = D\frac{d^2C}{dx^2} \quad \text{(S16)}$$

dC/dt is the rate of change of concentration over time (mol/mL/s), *D* is the diffusion coefficient of the chemical (cm²/s). To solve the diffusion equation into the well plate plastic numerically, we calculated the rate of concentration changes for the *i*-th layer ($dC_{\text{well}}[\text{i}]/dt$) using the diffusion coefficient of the chemical in the well plastic (D_{well}, cm²/s) and the chemical concentrations in adjacent layers ($C_{\text{well}}[\text{i+1}]$ and $C_{\text{well}}[\text{i–1}]$):

$$\frac{dC_{\text{well}}[\text{i}]}{dt} = D_{\text{well}} \cdot \frac{C_{\text{well}}[\text{i+1}] - 2 \cdot C_{\text{well}}[\text{i}] + C_{\text{well}}[\text{i}-1]}{dx^2_{\text{well}}} \quad \text{(S17)}$$

dx_{well} is the layer thickness (cm), calculated as the total well plate plastic thickness divided by the number of layers used to spatially resolve well plate wall. The term $2 \cdot C_{\text{well}}[\text{i}]$ in this equation balances the influence of both neighboring layers according to approximate the second spatial derivative of the concentration. Zero flux to outside the system is assumed at the outermost

layer.

Section S-3. Details on Lowry and the sulpho-phosphovanillin (SPV) assays.

For the Lowry assay, media (base medium or serum) were diluted in bidistilled water to match the BSA calibration range (0.1-1 mg/mL). In a 96-well plate, 200 µL of Lowry reagent (1% $CuSO_4$ in H_2O, 2% K−Na-tartrate in H_2O, 2% Na_2CO_3 in H_2O) was added to 20 µL of BSA standard or sample (Sigma). After vortexing for 5 min, 20 µL of 1 M Folin−Ciocalteu reagent (Sigma) was added. Following a 30 min incubation, absorbance at 750 nm was measured using an UV/Vis plate reader. Protein content was calculated using the BSA calibration curve. Prior to the SPV assay, lipids in the media were extracted following a modified Bligh-Dyer method.[7] Samples were mixed with 2-propanol and cyclohexane, then vortexed, sonicated, and centrifuged. The upper cyclohexane layer was collected and the extraction repeated. The cyclohexane was gathered, dried under nitrogen, treated with sulfuric acid, heated, and then reacted with phosphoric acid-vanillin reagent (Merck). Absorbance at 530 nm was measured, using triolein (Sigma), POPC (Avanti Polar Lipids), and cholesterol (Sigma) as calibration standards. The lipid content in media was calculated based on POPC, triolein, and cholesterol calibrations (0-100 µg/mL).

Section S-4. Details on Cellular Uptake Kinetics Experiments with PFAS.

Uptake kinetics of per- and polyfluoroalkyl substances (PFAS) were measured in HepG2 cells seeded at a density of 3.0×10^5 cells/well in 0.8 mL DMEM containing 0.5% FBS. Cells were allowed to attach for 12 h at 37 °C in a humidified incubator (5% CO_2, 90% RH). Exposure media were prepared in DMEM with 0.5% FBS and spiked with a mixture of four PFAS (perfluorooctanoic acid (PFOA), perfluorooctane sulfonic acid (PFOS), perfluorononanoic acid (PFNA), and perfluorododecanoic acid (PFDoDA)) at nominal concentrations of 2 µg/L. Spiked media were equilibrated for 2 h at 37 °C on an orbital shaker (100 rpm) prior to dosing to ensure complete dissolution and protein binding. Cells were dosed by adding 200 µL of PFAS-spiked medium per well, yielding a final medium volume of 1 mL.

Media and cells were harvested at 0, 10 min, 1, 2, 4, 8, 24, 40, and 48 h post-dosing. At each time point, medium was collected, vortexed, and spiked with mass-labeled internal standards (^{13}C-labeled analogues of each target PFAS, Wellington Laboratories) prior to extraction using acetonitrile and 1% formic acid in methanol. Supernatants were concentrated under nitrogen and reconstituted in injection standard (^{18}O-labeled PFAS analogues, Wellington Laboratories). Cells were washed with PBS to remove residual extracellular PFAS, detached with trypsin, and extracted via acetonitrile-based protein precipitation. Cell and medium extracts were analyzed using a SCIEX ExionLC AC UHPLC system coupled to a SCIEX X500R quadrupole time-of-flight tandem mass spectrometer (QTOF-MS/MS). Analytical methods and quality assurance/quality control (QAQC) procedures followed EPA Method 1633 and have been described in detail recently.[8]

Cell growth factors during uptake experiments were quantified using phase-contrast microscopy (Thermo EVOS M7000) and a custom *R* workflow based on the *EBImage* package. Images were background-corrected, thresholded, and morphologically processed to segment cell-covered from cell-free areas. Growth was expressed as fractional cell-covered area per well. The *R* script is provided below.

<u>*R* workflow for imaging of cell proliferation in PFAS experiments.</u>

```
library(EBImage)
library(openxlsx)

# Folder with images
img_dir <- "."              # current directory
pattern <- ".TIF"           # file extension

# Number of images to process
n_subset <- 100             # Number of images randomly selected

# Output Excel file
excel_out <- "cell_area_results.xlsx"

# Optional: save masks for QC
save_masks <- FALSE
mask_dir <- "masks"

# SEGMENTATION PARAMETERS (LOCKED)
bg_radius    <- 61
sigma        <- 1.5
```

```
otsu_scale   <- 0.75
open_radius  <- 3
close_radius <- 7

# Setup
files <- list.files(img_dir, pattern = pattern, full.names = TRUE)

if (length(files) == 0) {
  stop("No image files found in directory.")
}

# --- RANDOM SUBSET SELECTION ---
set.seed(123)   # remove or change seed if you want a different random subset
files <- sample(files, size = min(n_subset, length(files)))

# Save which images were used
write.csv(
  data.frame(file = basename(files)),
  "subset_used.csv",
  row.names = FALSE
)

if (save_masks) {
  dir.create(mask_dir, showWarnings = FALSE)
}

results <- data.frame(
  file = character(),
  cell_area_px = numeric(),
  frac_area = numeric(),
  stringsAsFactors = FALSE
)


# Batch loop
for (f in files) {

  img <- readImage(f)

  if (colorMode(img) != 0) {
    img <- channel(img, "gray")
  }

  # Background correction
  img.bg <- opening(img, makeBrush(bg_radius, "disc"))
  img.corrected <- img - img.bg
  img.corrected[img.corrected < 0] <- 0

  # Smoothing
  img.smooth <- gblur(img.corrected, sigma = sigma)

  # Thresholding
  th <- otsu(img.smooth)
  mask <- img.smooth > (th * otsu_scale)
```

```
  # Morphological cleanup
  mask <- opening(mask, makeBrush(open_radius, "disc"))
  mask <- closing(mask, makeBrush(close_radius, "disc"))
  mask <- fillHull(mask)

  # Quantification
  cell_area_px <- sum(mask)
  frac_area <- cell_area_px / prod(dim(mask))

  # Store results
  results <- rbind(
    results,
    data.frame(
      file = basename(f),
      cell_area_px = cell_area_px,
      frac_area = frac_area
    )
  )

  # Optional: save mask for QC
  if (save_masks) {
    writeImage(mask, file.path(mask_dir, basename(f)))
  }
}

# Write Excel file
write.xlsx(results, excel_out, overwrite = TRUE)

cat("Done.\n")
cat("Processed images:", nrow(results), "\n")
cat("Results written to:", excel_out, "\n")
```

Section S-5. Additional plots.

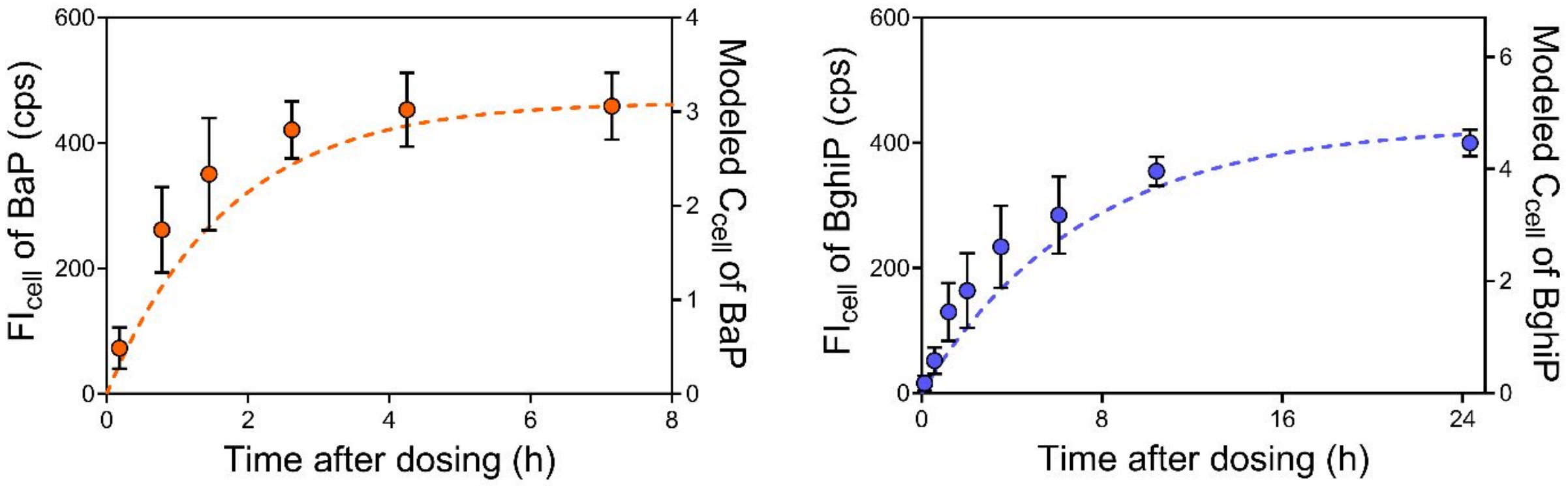

Figure S1. Cellular uptake kinetics of benzo[a]pyrene (BaP, left) and benzo[ghi]perylene (BghiP, right) following dosing. Symbols are experimental fluorescence intensities FI_{cell} (mean±SD) from Fischer et al. (2018),[9] and dashed lines are simulated cellular concentrations using the kinetic model with the chemical input parameters from Table S1. Simulations were run for 96-well plates containing 120 μL of DMEM with 0.5% FBS and 16,000 HEK293T cells to replicate assay conditions used in Fischer et al.[9]

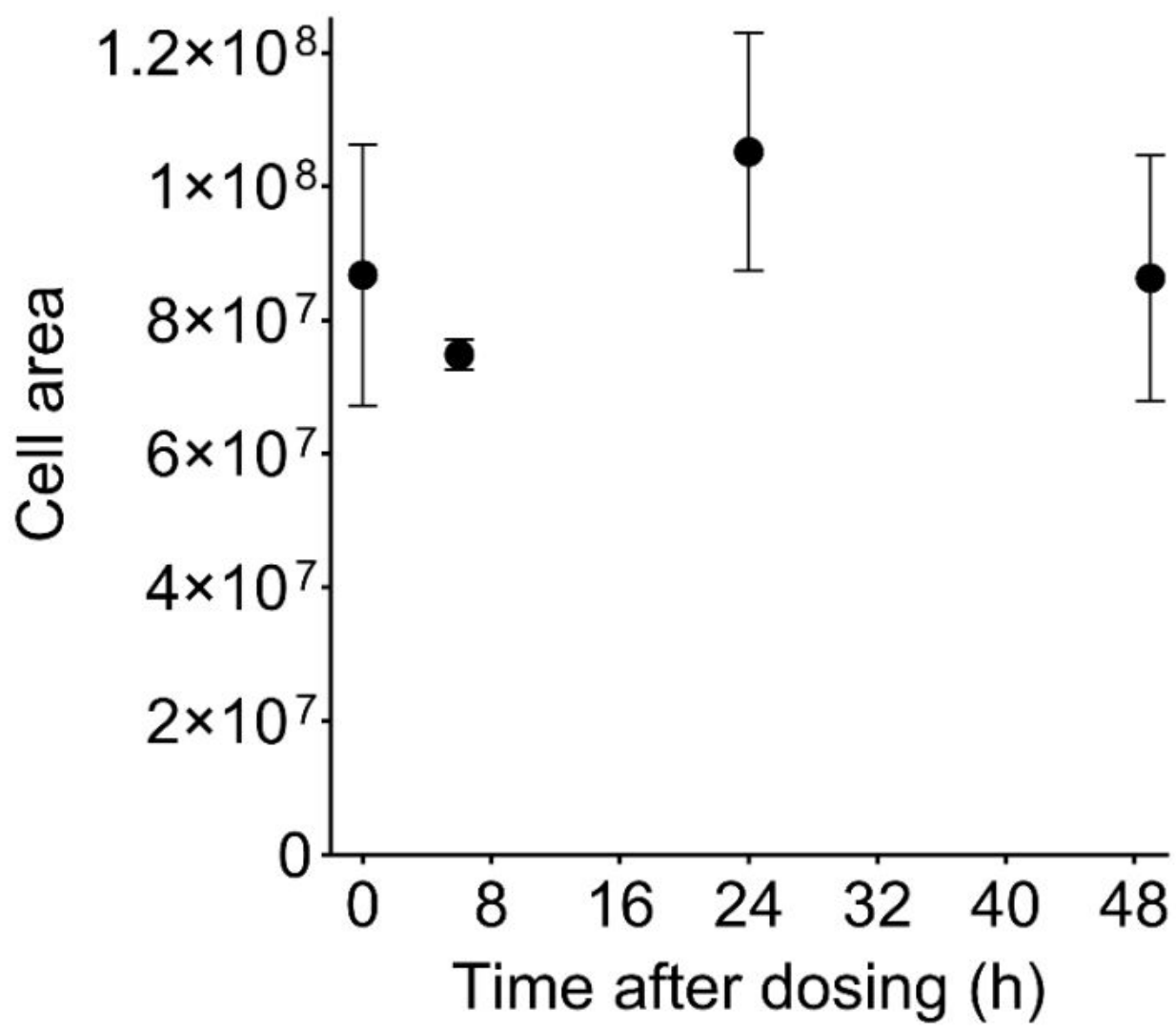

Figure S2. Image-based quantification of HepG2 cell pixel area over time following PFAS dosing in 6-well plates. Cellular area was determined from phase-contrast images acquired using a Thermo Fisher EVOS M7000 microscope and quantified by automated segmentation of cellular versus non-cellular regions. Data points represent mean cell-covered pixel area per well, with error bars indicating variability across three wells per time point (n = 3).

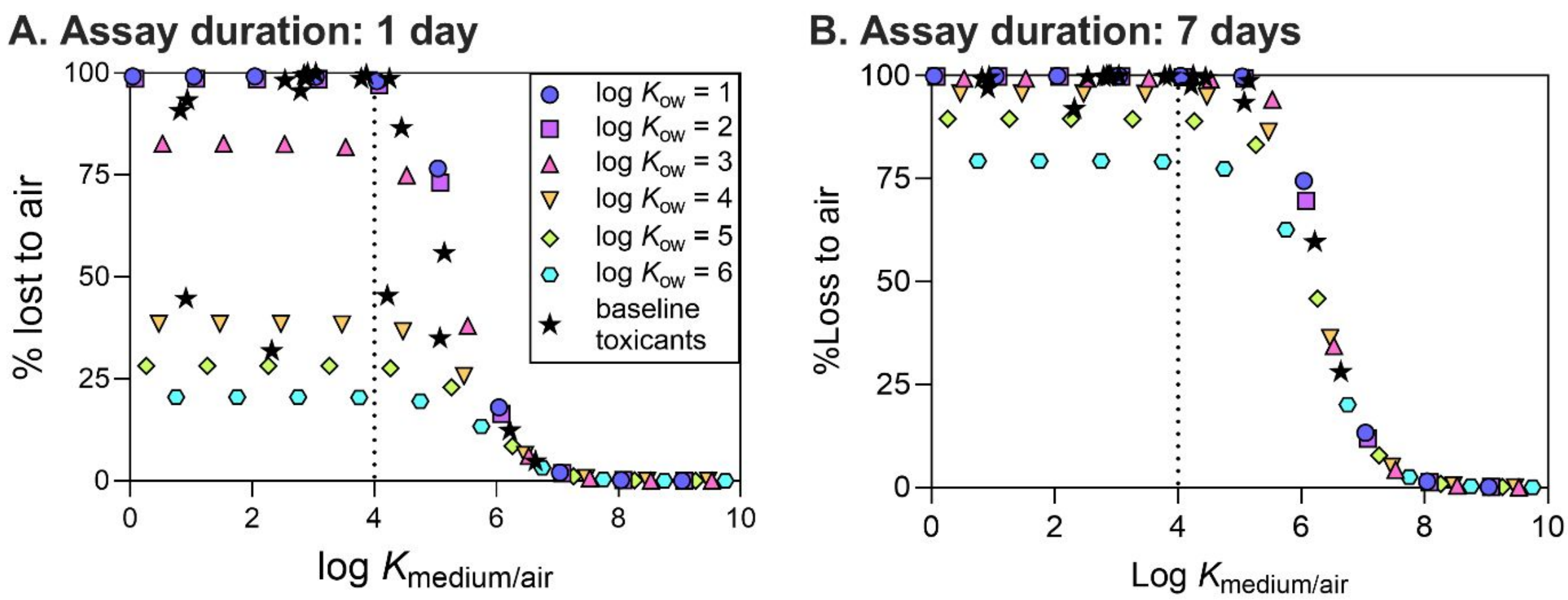

Figure S3. (A) Simulated percent loss to air over 24 h versus medium-air partition coefficients ($K_{medium/air}$) for 126 fictional chemicals and 19 baseline toxicants (see **Table S8** for chemical properties). Simulations were done for 5,000 HepG2 cells in 384-well plates and medium with 10% FBS. The dashed line shows the empirical volatility cut-off. **(B)** Simulated percent loss to air over 7 days versus medium-air partition coefficients ($K_{medium/air}$) for 126 fictional chemicals and 19 baseline toxicants (see **Table S8** for chemical properties). Simulations were done for 5,000 HepG2 cells in 384-well plates and medium with 10% FBS. The dashed line shows the empirical volatility cut-off.

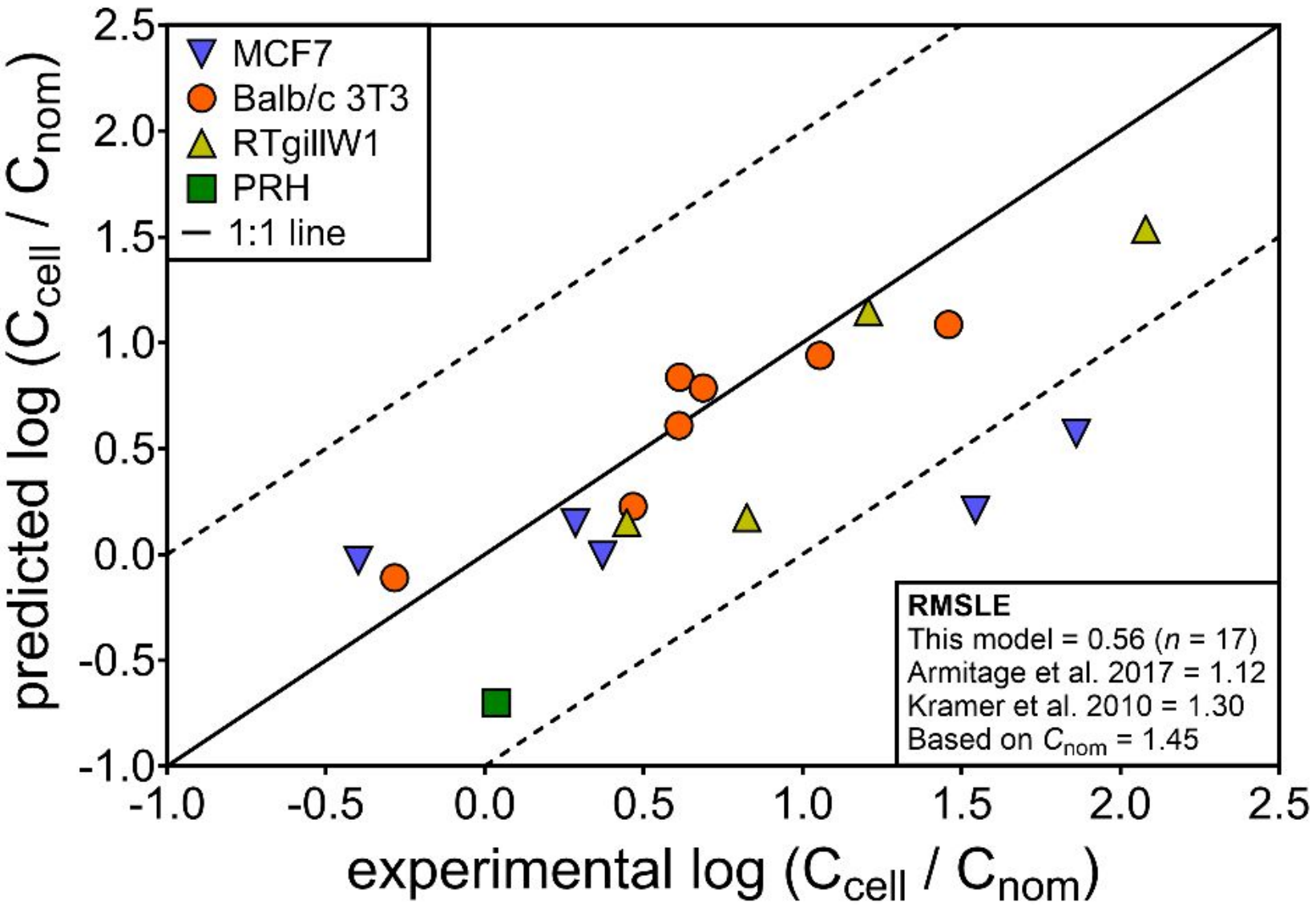

Figure S4. Predicted versus experimental intracellular concentrations (log (C_{cell}/C_{nom})) for 17 neutral chemicals across four cell lines (Balb/c 3T3, RTgill-W1, primary rat hepatocytes (PRH), and MCF-7 cells.[10-13] The solid line indicates 1:1 agreement and dashed lines indicate one order of magnitude error bounds. Model performance (RMSLE = 0.56) is compared to predictions based on nominal concentrations and two previously published models.[11,14,15] See **Table S10** for underlying data.

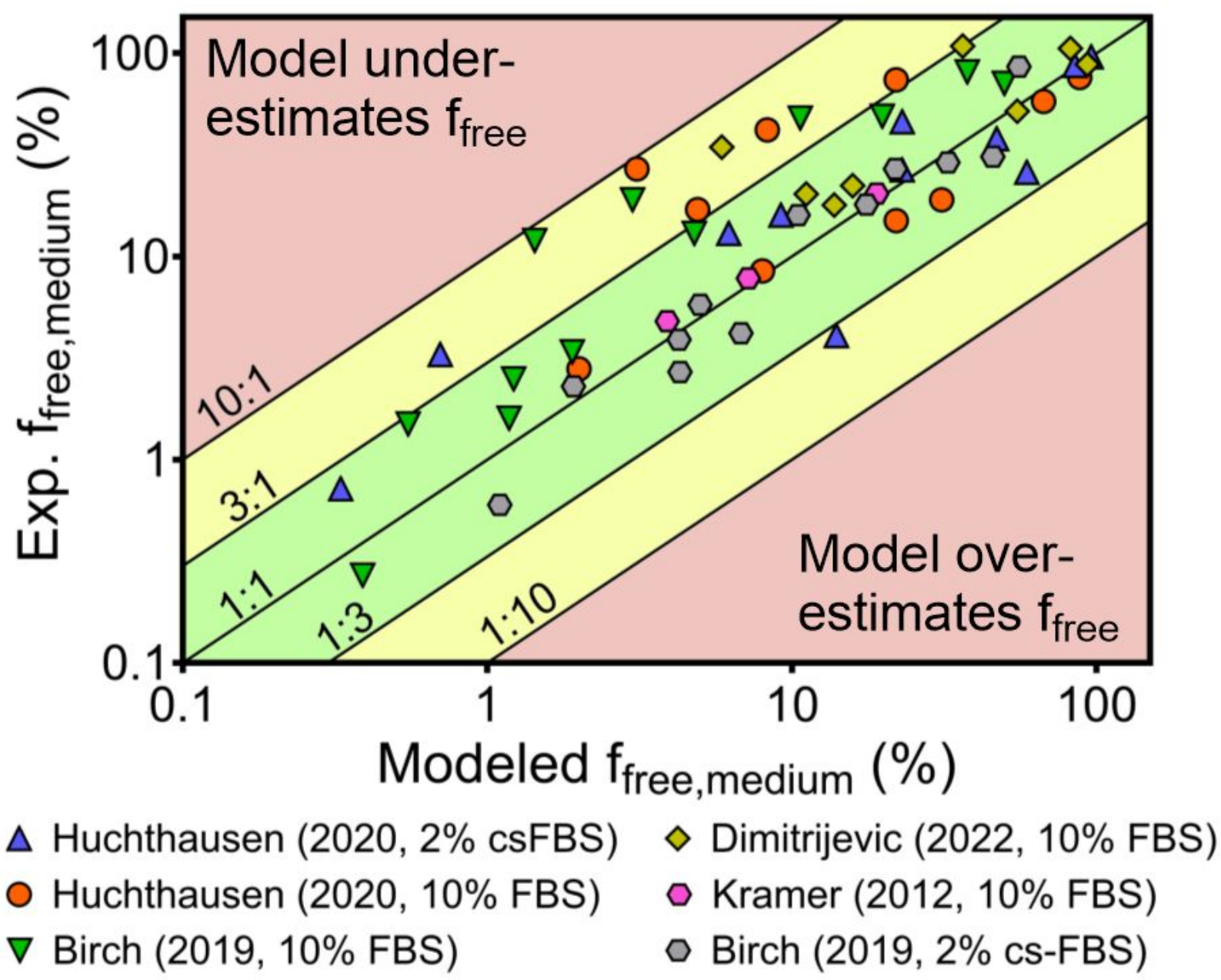

Figure S5. Modeled vs. experimental free fractions in media (%) after 24 hours of incubation across multiple chemicals and assay setups (see **Table S9** for details).[10,15-20]

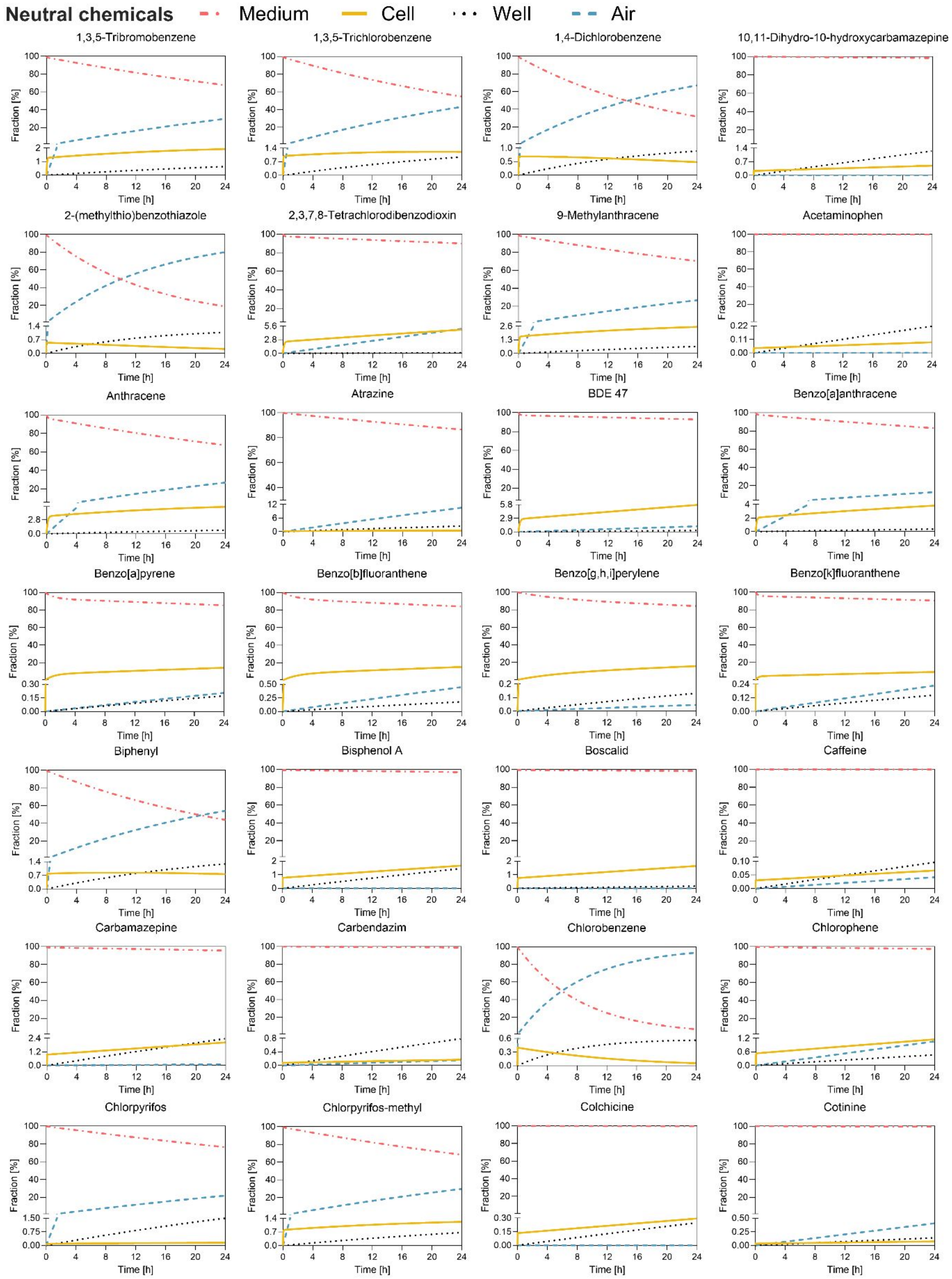

Figure S6. Individual predictions of fractions (%) in medium, cell, well, and air over for neutral compounds over 24 hours (part 1). Simulations were performed for 5000 HepG2 cells exposed to 40 µL of DMEM supplemented with 10% FBS.

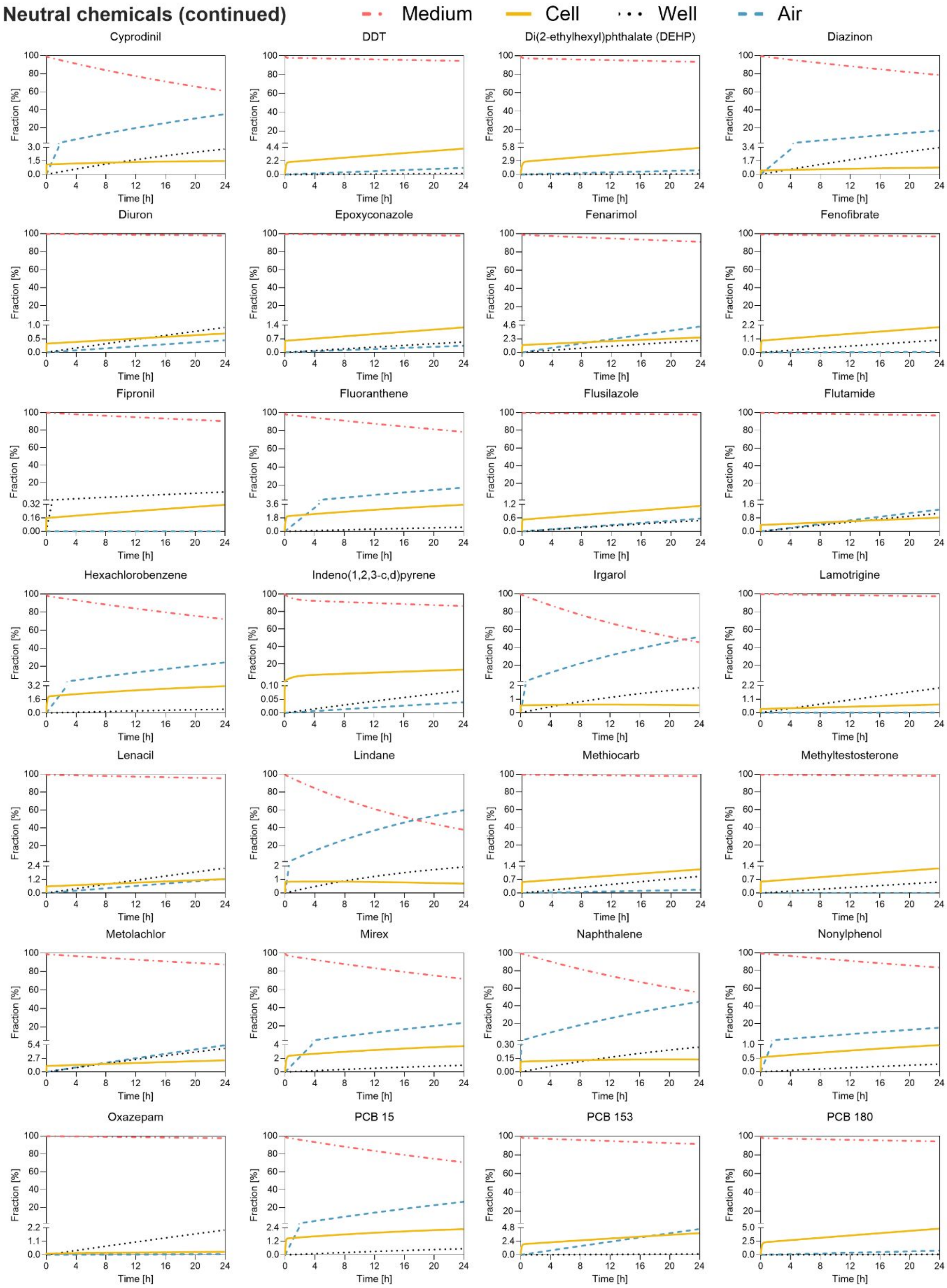

Figure S7. Individual predictions of fractions (%) in medium, cell, well, and air over for neutral compounds over 24 hours (part 2). Simulations were performed for 5000 HepG2 cells exposed to 40 µL of DMEM supplemented with 10% FBS.

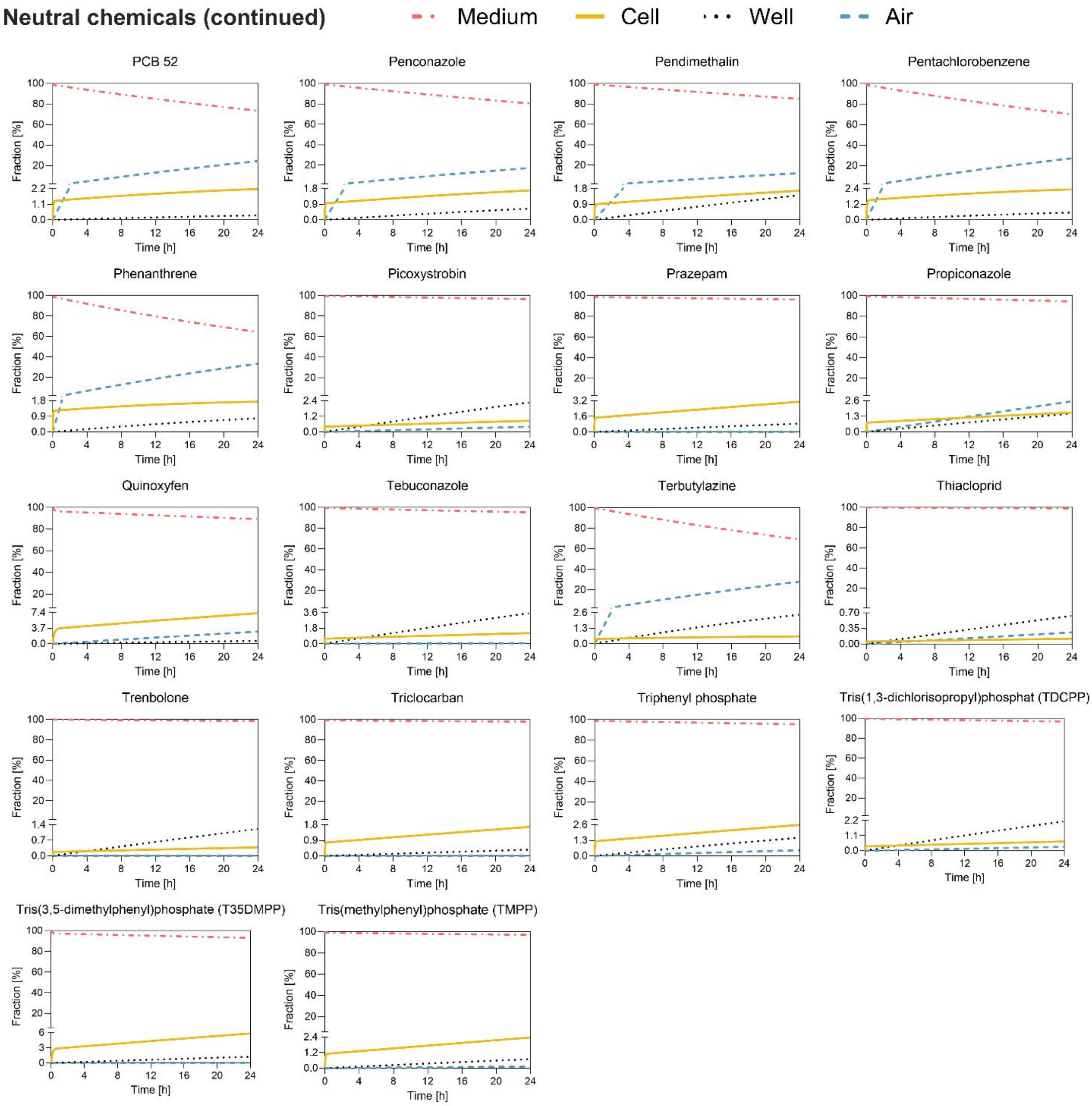

Figure S8. Individual predictions of fractions (%) in medium, cell, well, and air over for neutral compounds over 24 hours (part 3). Simulations were performed for 5000 HepG2 cells exposed to 40 µL of DMEM supplemented with 10% FBS.

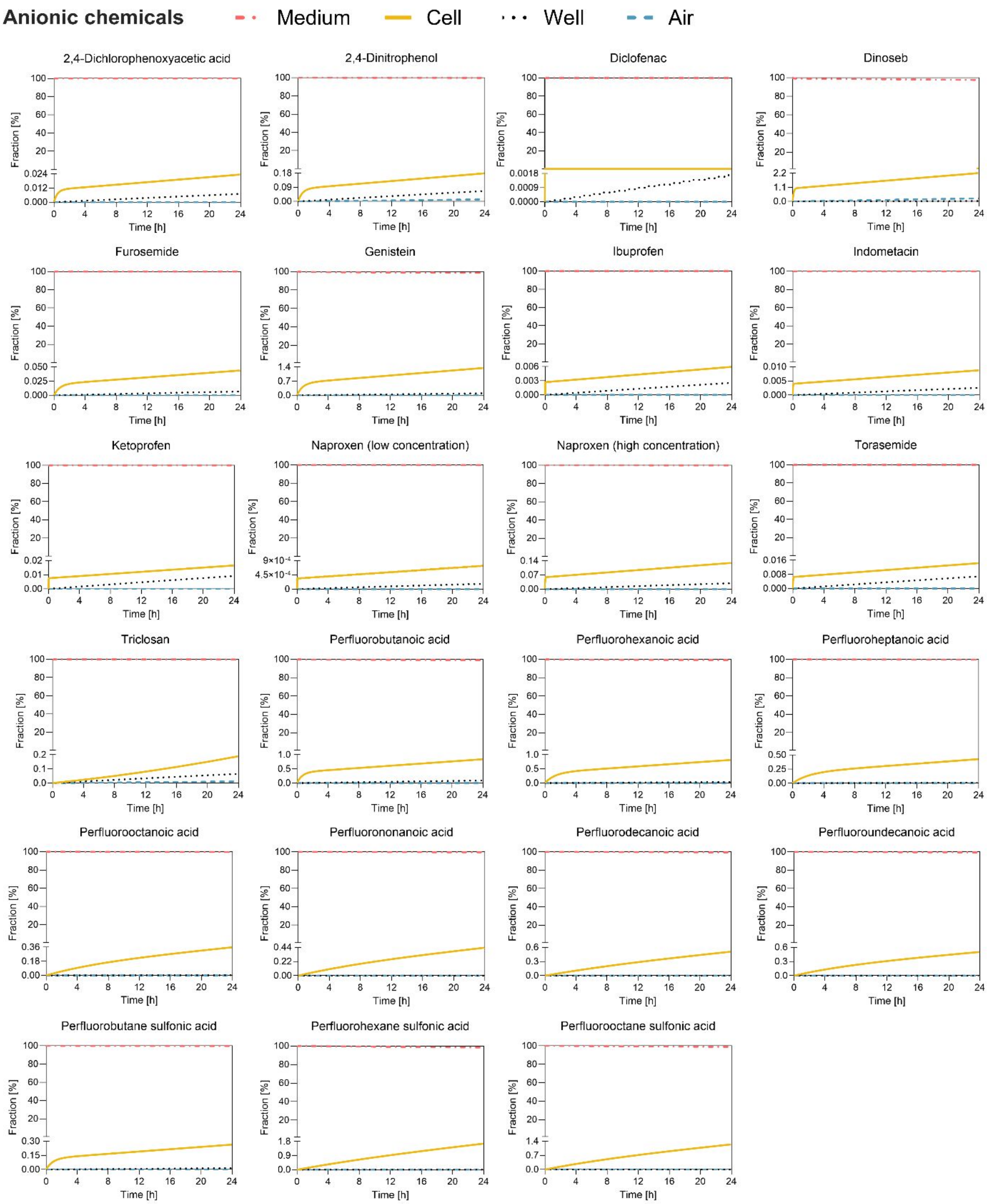

Figure S9. Individual predictions of fractions (%) in medium, cell, well, and air over for anionic compounds over 24 hours. Simulations were performed for 5000 HepG2 cells exposed to 40 μL of DMEM supplemented with 10% FBS.

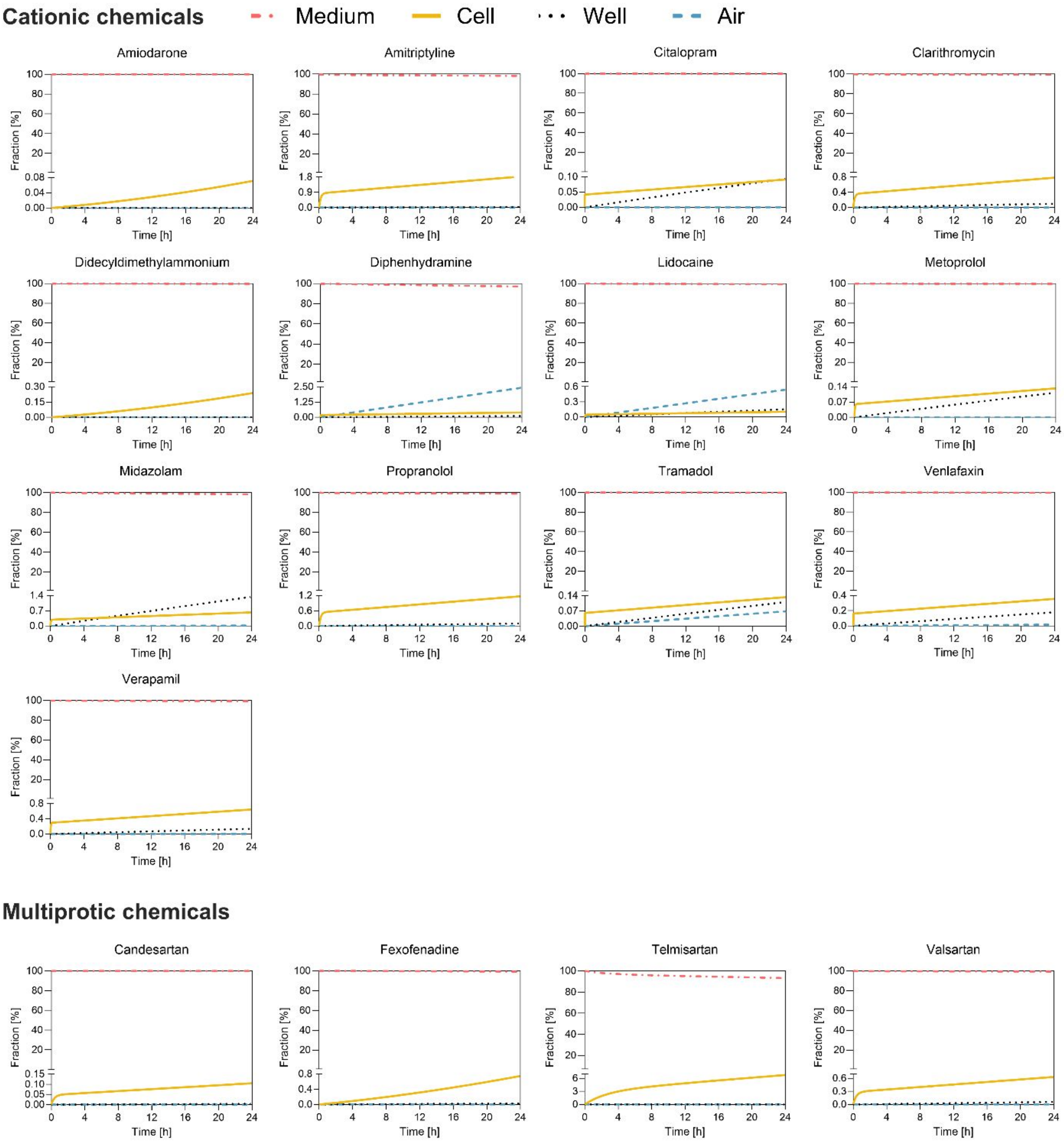

Figure S10. Individual predictions of fractions (%) in medium, cell, well, and air over for cationic and multiprotic compounds over 24 hours. Simulations were performed for 5000 HepG2 cells exposed to 40 µL of DMEM supplemented with 10% FBS.

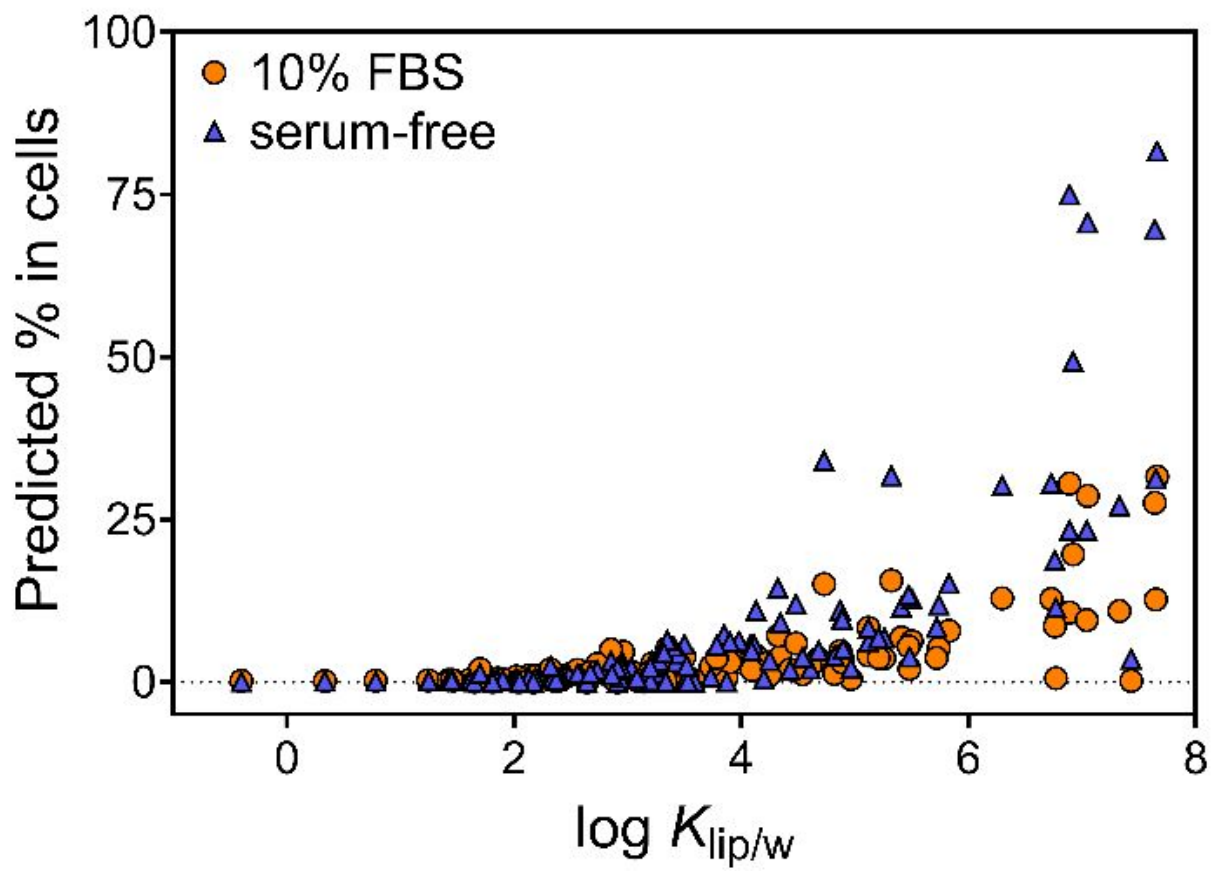

Figure S11. Predicted percent of total chemical mass in HepG2 cells (n = 2,000) after 24 h exposure in 1536-well format as a function of log $K_{lip/w}$. Simulations were performed under serum-free and 10% FBS conditions. Addition of FBS reduced cellular uptake for lipophilic compounds (log $K_{lip/w}$ > 4) due to increased binding to medium constituents, illustrating the buffering effect of serum proteins and lipids.

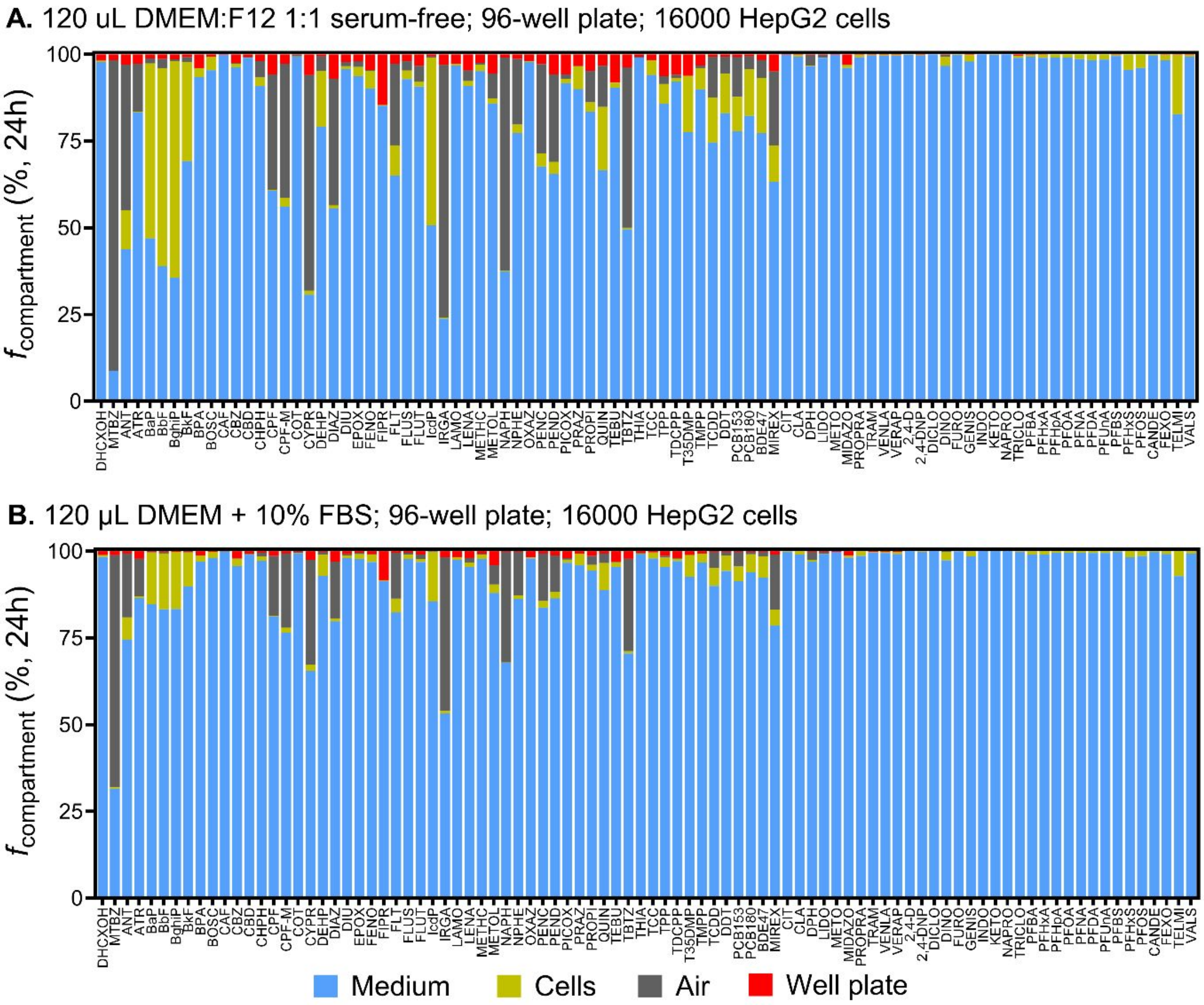

Figure S12. Simulated fractions of 100 of 113 chemicals (names, abbreviations and physicochemical properties in **Table S1**) across compartments ($f_{compartment}$) in 96-well plate assays after 24 h of incubation. Thirteen chemicals were randomly excluded for visual clarity. Simulations were performed for 16000 HepG2 cells exposed to 120 µL of either serum-free DMEM:F12 (1:1) (A) or DMEM supplemented with 10% FBS (B).

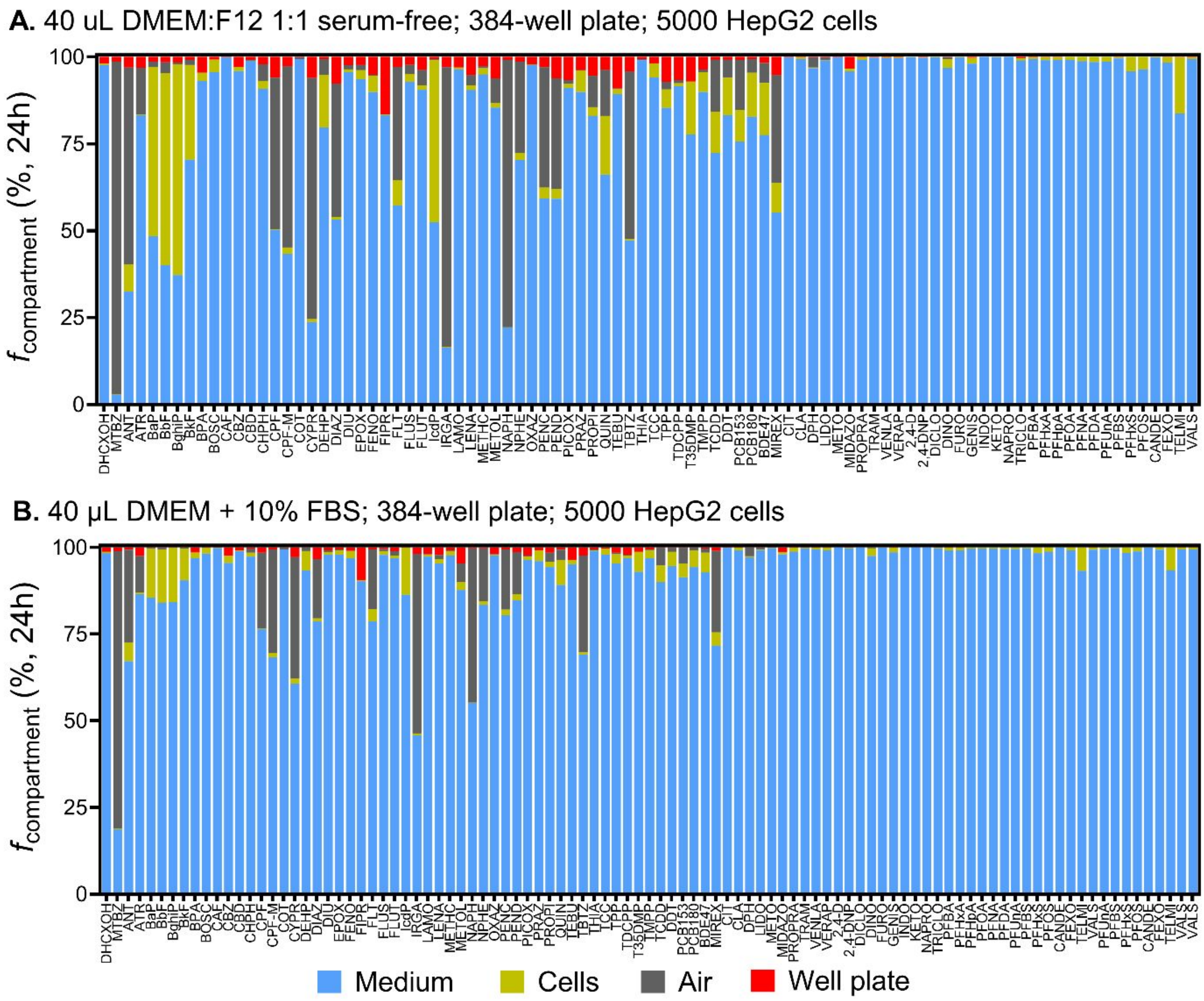

Figure S13. Simulated fractions of 100 of 113 chemicals (names, abbreviations and physicochemical properties in **Table S1**) across compartments ($f_{compartment}$) in 384-well plate assays after 24 h of incubation. Thirteen chemicals were randomly excluded for visual clarity. Simulations were performed for 5000 HepG2 cells exposed to 40 µL of (A) serum-free DMEM:F12 (1:1) or (B) DMEM supplemented with 10% FBS.

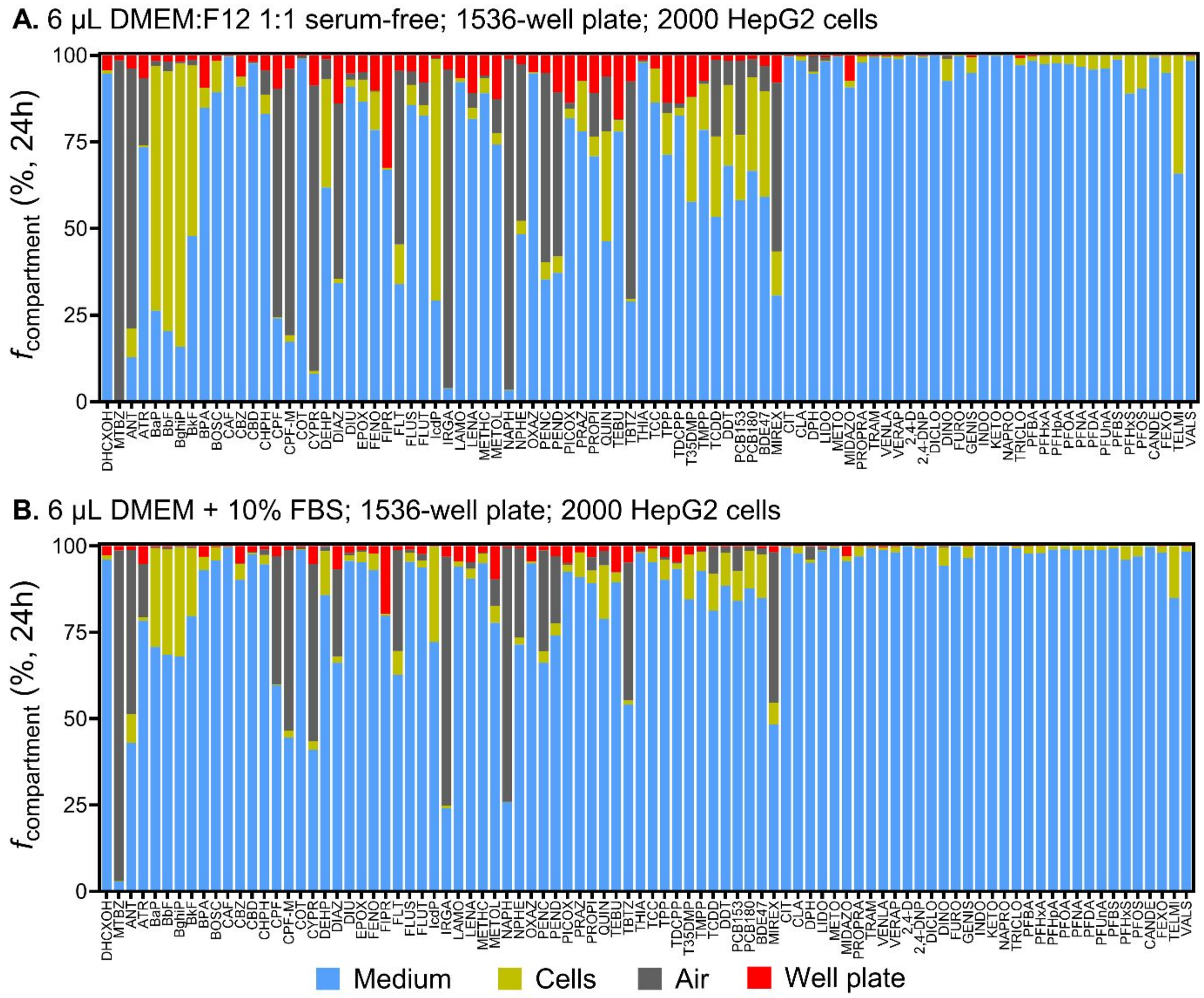

Figure S14. Simulated fractions of 100 of 113 chemicals (names, abbreviations and physicochemical properties in **Table S1**) across compartments ($f_{compartment}$) in 1536-well plate assays after 24 h of incubation. Simulations were performed for 2000 HepG2 cells exposed to 6 µL of either serum-free DMEM:F12 (1:1) (A) or DMEM supplemented with 10% FBS (B).

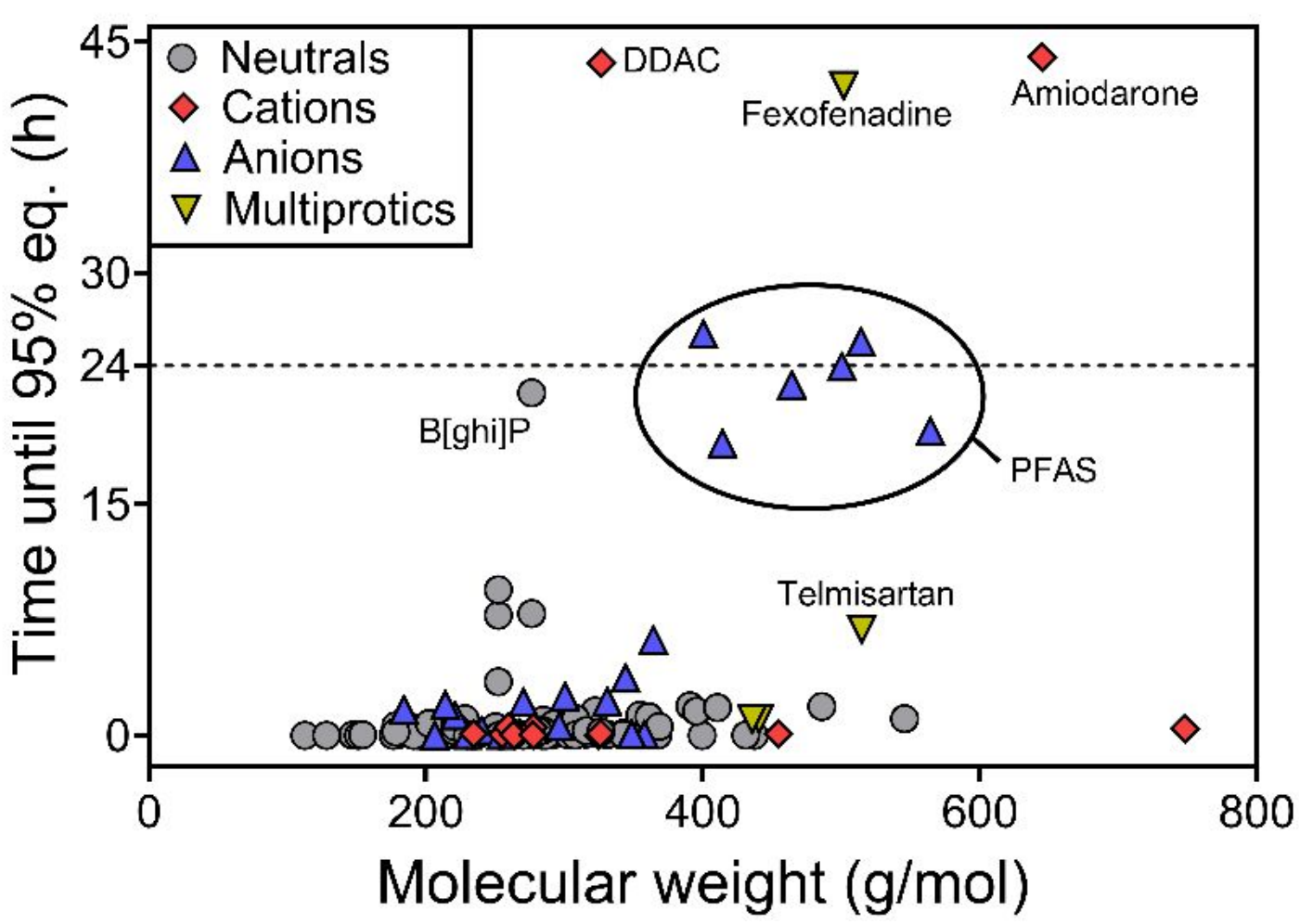

Figure S15. Simulated time required to reach 95% of equilibrium intracellular concentrations as a function of molecular weight for 113 chemicals, classified by predominant ionization. The model was run using a 384-well plate with 5,000 HepG2 cells and serum-free DMEM:F12 (1:1).

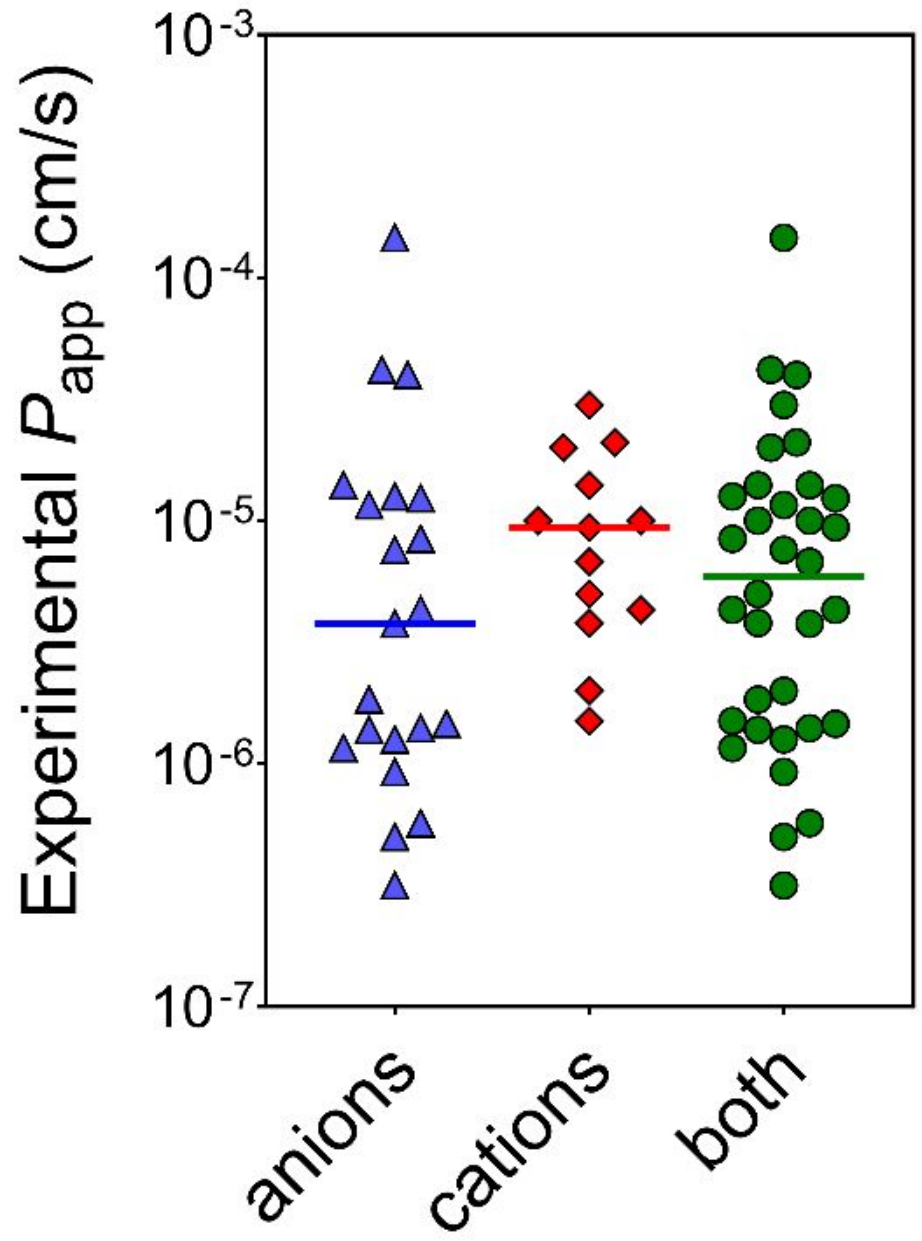

Figure S16. Experimental apparent permeability coefficients (P_{app}) of ionizable organic chemicals, collected from the literature and summarized in **Table S1**, grouped by dominant charge state at pH 7.4: anions (blue triangles), cations (red diamonds), and all IOCs combined (green circles). Horizontal lines indicate the median of each group. The median across all IOCs was approximately $5x10^{-6}$ cm/s and can be used as an estimate for IOCs in the model.

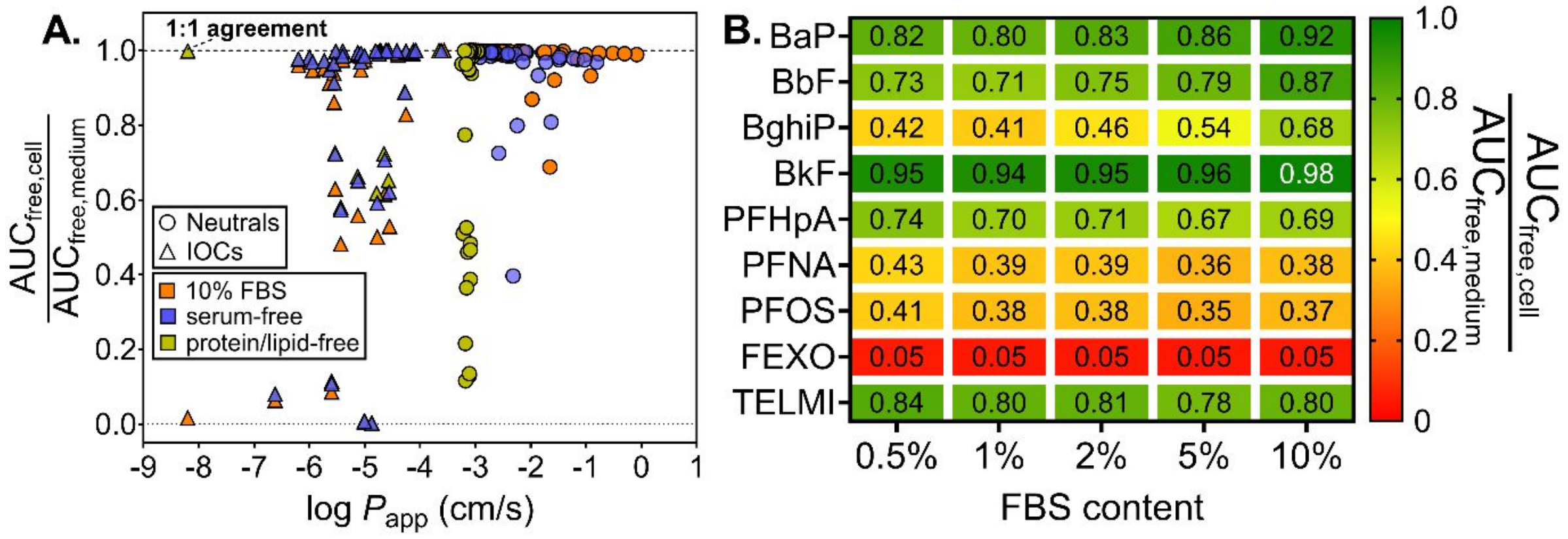

Figure S17. (A) Ratio of $AUC_{free,cell}$ to $AUC_{free,medium}$ over 24 h as a function of log apparent permeability (P_{app}) for 113 compounds simulated in a 384-well plate (40 µL medium, 5,000 HepG2 cells) under three medium conditions. Ratios below 1 indicate that the equilibrium model may overestimate cellular exposure within a standard 24 h assay window assuming $C_{free,cell} = C_{free,medium}$ at all times. **(B)** Ratio of $AUC_{free,cell}$ to $AUC_{free,medium}$ over 24 h for selected compounds across increasing FBS content, simulated under conditions from Fischer et al.[9] using a 96-well plate with 9,000-16,000 HEK293T cells and DMEM GlutaMAX supplemented with 0.5-10% FBS. Cell growth was adjusted based on FBS content (**Table S6**).

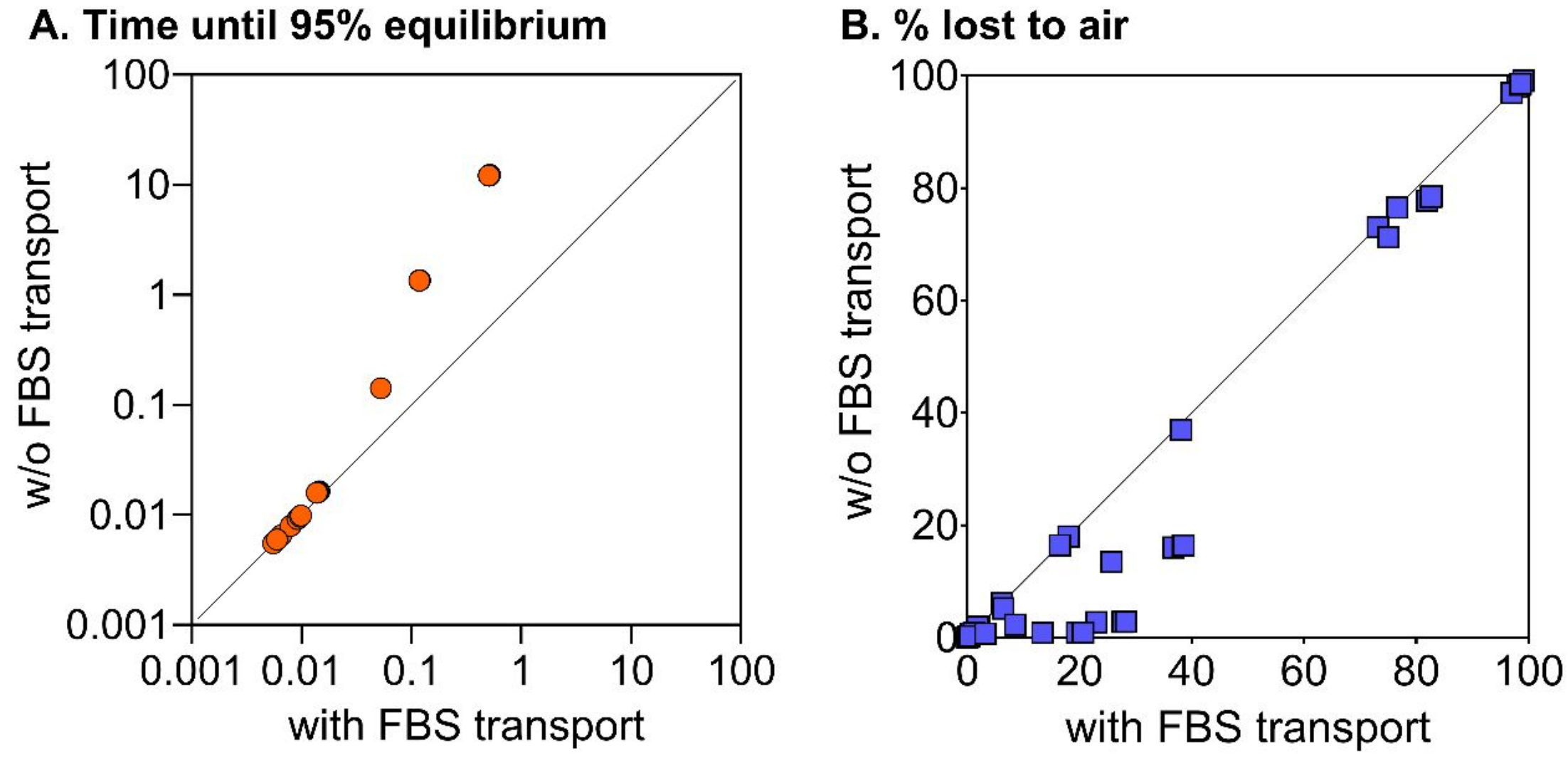

Figure S18. Effect of facilitated transport by FBS-bound chemicals across the UWL on (A) time to reach 95% of intracellular equilibrium ($t_{95\%}$, h) and (B) percent loss to air after 24 h. Simulations were performed with and without (w/o) facilitated transport for 126 hypothetical compounds in a 384-well plate (40 µL DMEM with 5,000 HepG2 cells). The diagonal lines indicate 1:1 agreement.

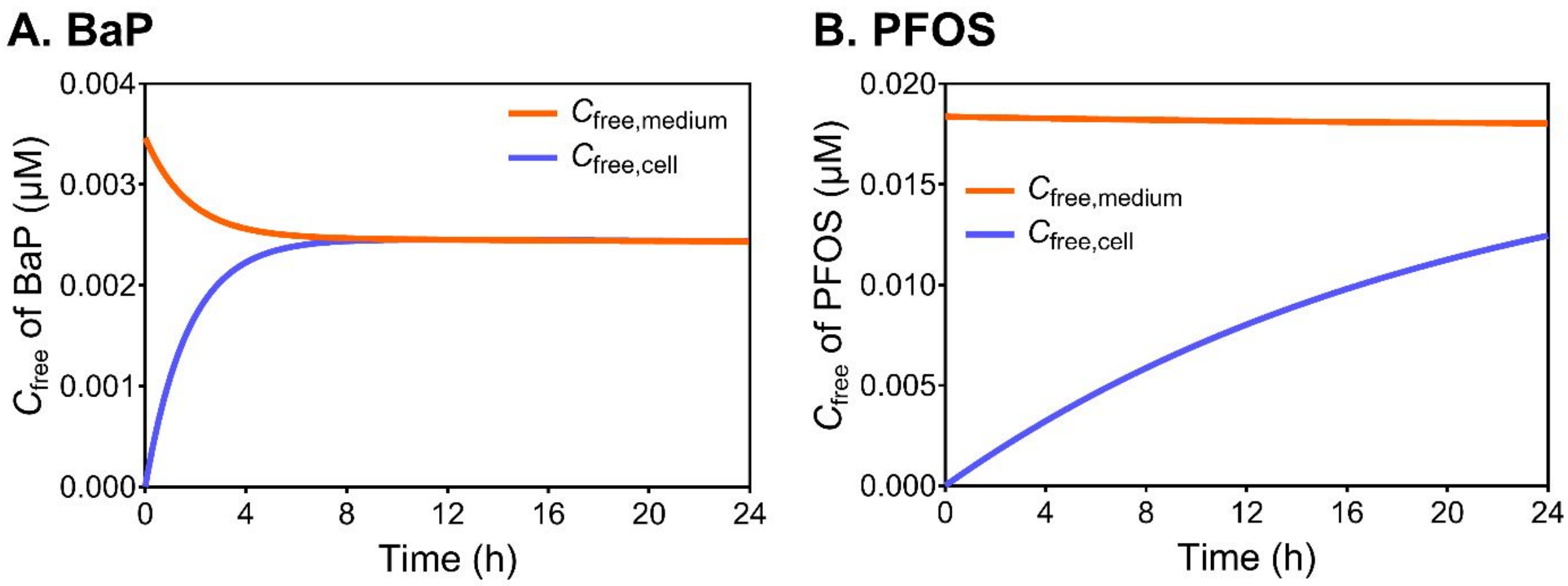

Figure S19. Simulated freely dissolved concentrations in medium ($C_{free,medium}$) and cells ($C_{free,cell}$) over 24 h for **(A)** benzo[a]pyrene (BaP) and **(B)** perfluorooctanesulfonic acid (PFOS). Simulations were performed for 16,000 HEK293 cells exposed to 120 µL of DMEM GlutaMAX supplemented with 0.5% FBS in a 96-well plate, replicating conditions from Fischer et al (2018).[9]

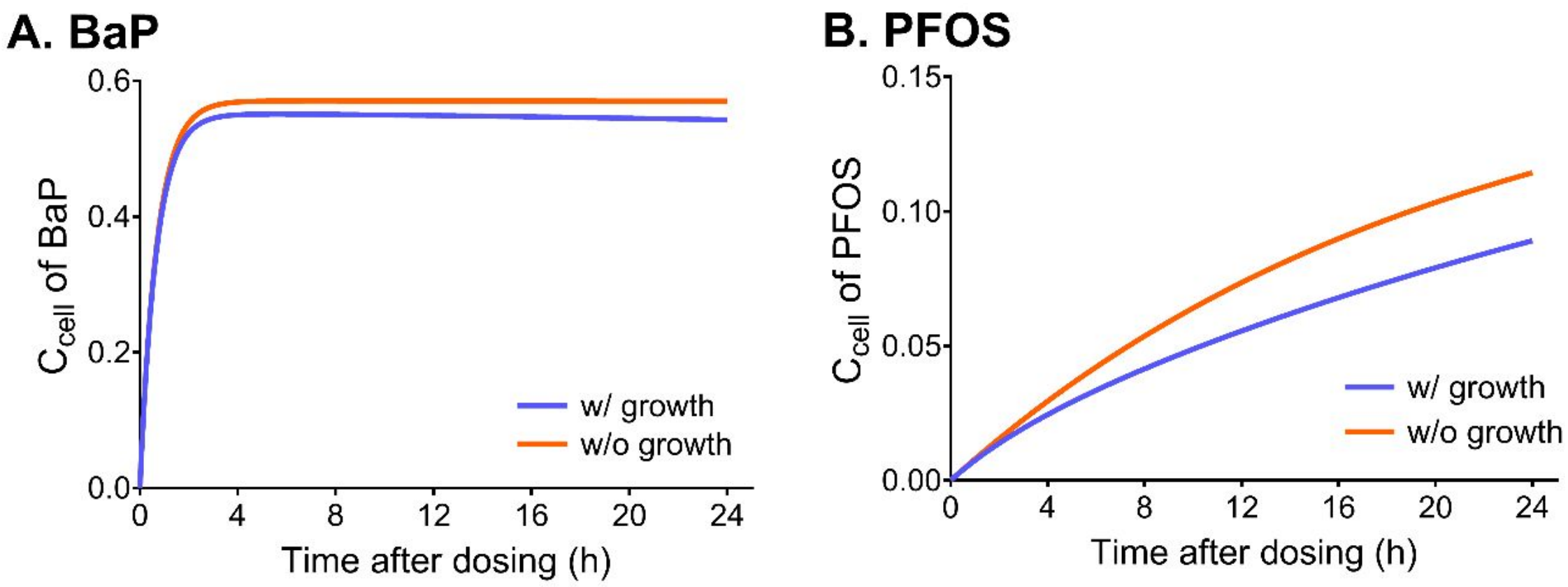

Figure S20. Simulated cellular concentration-time profiles for **(A)** benzo[a]pyrene (BaP) and **(B)** perfluorooctanesulfonic acid (PFOS) in a 96-well plate following exposure in DMEM GlutaMAX supplemented with 10% fetal bovine serum (FBS). Simulations compare conditions with cell growth (blue; cell number increasing from 16,000 cells at time 0h to 2.20x over 24h) and without cell growth (orange; constant 16,000 cells).

Section S-6. References used in the Supporting Information.

(1) Ter Laak, T. L.; Van Eijkeren, J. C. H.; Busser, F. J. M.; Van Leeuwen, H. P.; Hermens, J. L. M. Facilitated Transport of Polychlorinated Biphenyls and Polybrominated Diphenyl Ethers by Dissolved Organic Matter. *Environmental Science & Technology* **2009**, *43* (5), 1379-1385, Article. DOI: 10.1021/es802403v.
(2) ter Laak, T. L.; ter Bekke, M. A.; Hermens, J. L. M. Dissolved Organic Matter Enhances Transport of PAHs to Aquatic Organisms. *Environmental Science & Technology* **2009**, *43* (19), 7212-7217. DOI: 10.1021/es803684f.
(3) Fischer, F. C.; Cirpka, O. A.; Goss, K. U.; Henneberger, L.; Escher, B. I. Application of Experimental Polystyrene Partition Constants and Diffusion Coefficients to Predict the Sorption of Neutral Organic Chemicals to Multiwell Plates in in Vivo and in Vitro Bioassays. *Environ Sci Technol* **2018**, *52* (22), 13511-13522. DOI: 10.1021/acs.est.8b04246.
(4) Escher, B. I.; Glauch, L.; König, M.; Mayer, P.; Schlichting, R. Baseline Toxicity and Volatility Cutoff in Reporter Gene Assays Used for High-Throughput Screening. *Chemical Research in Toxicology* **2019**, *32* (8), 1646-1655. DOI: 10.1021/acs.chemrestox.9b00182.
(5) Fischer, F. C.; Henneberger, L.; Schlichting, R.; Escher, B. I. How To Improve the Dosing of Chemicals in High-Throughput in Vitro Mammalian Cell Assays. *Chemical Research in Toxicology* **2019**, *32* (8), 1462-1468. DOI: 10.1021/acs.chemrestox.9b00167.
(6) Tanneberger, K.; Rico-Rico, A.; Kramer, N. I.; Busser, F. J. M.; Hermens, J. L. M.; Schirmer, K. Effects of Solvents and Dosing Procedure on Chemical Toxicity in Cell-Based in Vitro Assays. *Environmental Science & Technology* **2010**, *44* (12), 4775-4781. DOI: 10.1021/es100045y.
(7) Fischer, F. C.; Henneberger, L.; König, M.; Bittermann, K.; Linden, L.; Goss, K.-U.; Escher, B. I. Modeling Exposure in the Tox21 in Vitro Bioassays. *Chemical Research in Toxicology* **2017**, *30* (5), 1197-1208. DOI: 10.1021/acs.chemrestox.7b00023.
(8) Agudelo Areiza, J.; Becanova, J.; Vojta, S.; Ganglbauer, J.; Apte, U.; Brunetti, L.; Kumer, S.; Haque, F.; Kim, E.; Sunderland, E. M.; Lohmann, R.; Fischer, F. C.; Slitt, A. Presence of Legacy and Emerging PFAS in Human Liver Specimens Banked in the United States from 2000 to 2024. *Environmental Science & Technology* **2026**, *60* (15), 11281-11292. DOI: 10.1021/acs.est.5c14780.
(9) Fischer, F. C.; Abele, C.; Droge, S. T. J.; Henneberger, L.; König, M.; Schlichting, R.; Scholz, S.; Escher, B. I. Cellular Uptake Kinetics of Neutral and Charged Chemicals in in Vitro

Assays Measured by Fluorescence Microscopy. *Chemical Research in Toxicology* **2018**, *31* (8), 646-657. DOI: 10.1021/acs.chemrestox.8b00019.
(10) Dimitrijevic, D.; Fabian, E.; Nicol, B.; Funk-Weyer, D.; Landsiedel, R. Toward Realistic Dosimetry In Vitro: Determining Effective Concentrations of Test Substances in Cell Culture and Their Prediction by an In Silico Mass Balance Model. *Chem Res Toxicol* **2022**, *35* (11), 1962-1973. DOI: 10.1021/acs.chemrestox.2c00128.
(11) Scherer, M. N.; Feshuk, M.; Armitage, J. M.; Arnot, J. A.; Crizer, D.; DeVito, M.; Ferguson, S. S.; Freeman, K.; Honda, G. S.; Harrill, J. A.; Sangion, A.; Tebes-Stevens, C.; Paul Friedman, K.; Wambaugh, J. F. Characterizing Accuracy of Model Predictions for Chemical Concentration in High Throughput Screening Assays. *Environmental Science & Technology* **2026**, *60* (6), 4869-4877. DOI: 10.1021/acs.est.5c10195.
(12) Stadnicka-Michalak, J.; Tanneberger, K.; Schirmer, K.; Ashauer, R. Measured and Modeled Toxicokinetics in Cultured Fish Cells and Application to In Vitro - In Vivo Toxicity Extrapolation. *Plos One* **2014**, *9* (3). DOI: 10.1371/journal.pone.0092303.
(13) Truisi, G. L.; Consiglio, E. D.; Parmentier, C.; Savary, C. C.; Pomponio, G.; Bois, F.; Lauer, B.; Jossé, R.; Hewitt, P. G.; Mueller, S. O.; Richert, L.; Guillouzo, A.; Testai, E. Understanding the biokinetics of ibuprofen after single and repeated treatments in rat and human in vitro liver cell systems. *Toxicol Lett* **2015**, *233* (2), 172-186. DOI: 10.1016/j.toxlet.2015.01.006.
(14) Armitage, J. M.; Wania, F.; Arnot, J. A. Application of Mass Balance Models and the Chemical Activity Concept To Facilitate the Use of in Vitro Toxicity Data for Risk Assessment. *Environmental Science & Technology* **2014**, *48* (16), 9770-9779. DOI: 10.1021/es501955g.
(15) Kramer, N. I.; Krismartina, M.; Rico-Rico, A.; Blaauboer, B. J.; Hermens, J. L. M. Quantifying Processes Determining the Free Concentration of Phenanthrene in Basal Cytotoxicity Assays. *Chemical Research in Toxicology* **2012**, *25* (2), 436-445. DOI: 10.1021/tx200479k.
(16) Huchthausen, J.; König, M.; Escher, B. I.; Henneberger, L. Experimental exposure assessment for in vitro cell-based bioassays in 96- and 384-well plates. *Front Toxicol* **2023**, *5*, 1221625. DOI: 10.3389/ftox.2023.1221625.
(17) Henneberger, L.; Huchthausen, J.; König, M.; Menge, A.; Wojtysiak, N.; Escher, B. I. Experimental exposure assessment of designed chemical mixtures in cell-based in vitro bioassays. *Frontiers in Environmental Chemistry* **2022**, *3*, Original Research. DOI: 10.3389/fenvc.2022.1018162.
(18) Henneberger, L.; Muhlenbrink, M.; Heinrich, D. J.; Teixeira, A.; Nicol, B.; Escher, B. I. Experimental Validation of Mass Balance Models for in Vitro Cell-Based Bioassays. *Environ Sci Technol* **2020**, *54* (2), 1120-1127. DOI: 10.1021/acs.est.9b06144.
(19) Henneberger, L.; Muhlenbrink, M.; Konig, M.; Schlichting, R.; Fischer, F. C.; Escher, B. I. Quantification of freely dissolved effect concentrations in in vitro cell-based bioassays. *Arch Toxicol* **2019**, *93* (8), 2295-2305. DOI: 10.1007/s00204-019-02498-3.
(20) Birch, H.; Kramer, N. I.; Mayer, P. Time-Resolved Freely Dissolved Concentrations of Semivolatile and Hydrophobic Test Chemicals in In Vitro Assays—Measuring High Losses and Crossover by Headspace Solid-Phase Microextraction. *Chemical Research in Toxicology* **2019**, *32* (9), 1780-1790. DOI: 10.1021/acs.chemrestox.9b00133.
